# AHL-Priming Protein 1 mediates *N*-3-oxo-tetradecanoyl-homoserine lactone priming in Arabidopsis

**DOI:** 10.1186/s12915-022-01464-3

**Published:** 2022-12-05

**Authors:** Abhishek Shrestha, Casandra Hernández-Reyes, Maja Grimm, Johannes Krumwiede, Elke Stein, Sebastian T. Schenk, Adam Schikora

**Affiliations:** 1 Julius Kühn Institute (JKI)—Federal Research Centre for Cultivated Plants, Institute for Epidemiology and Pathogen Diagnostics, Messeweg 11/12, 38104 Braunschweig, Germany; 2 Cell Biology, Faculty of Biology, University of Freiburg, 79104 Freiburg, Germany; 3 Justus Liebig University Giessen, Institute for Phytopathology, , Heinrich-Buff-Ring 26, 35392 Giessen, Germany

**Keywords:** AHL priming, Enhanced resistance, *N*-acyl homoserine lactone, Plant immunity, Plant–microbe interaction, Quorum sensing

## Abstract

**Background:**

*N*-3-oxo-tetradecanoyl-*L*-homoserine lactone (oxo-C14-HSL) is one of the *N*-acyl homoserine lactones (AHL) that mediate quorum sensing in Gram-negative bacteria. In addition to bacterial communication, AHL are involved in interactions with eukaryotes. Short-chain AHL are easily taken up by plants and transported over long distances. They promote root elongation and growth. Plants typically do not uptake hydrophobic long sidechain AHL such as oxo-C14-HSL, although they prime plants for enhanced resistance to biotic and abiotic stress. Many studies have focused on priming effects of oxo-C14-HSL for enhanced plant resistance to stress. However, specific plant factors mediating oxo-C14-HSL responses in plants remain unexplored. Here, we identify the Arabidopsis protein ALI1 as a mediator of oxo-C14-HSL-induced priming in plants.

**Results:**

We compared oxo-C14-HSL-induced priming between wild-type Arabidopsis Col-0 and an oxo-C14-HSL insensitive mutant *ali1*. The function of the candidate protein ALI1 was assessed through biochemical, genetic, and physiological approaches to investigate if the loss of the *ALI1* gene resulted in subsequent loss of AHL priming. Through different assays, including MAP kinase activity assay, gene expression and transcriptome analysis, and pathogenicity assays, we revealed a loss of AHL priming in *ali1*. This phenomenon was reverted by the reintroduction of *ALI1* into *ali1*. We also investigated the interaction between ALI1 protein and oxo-C14-HSL using biochemical and biophysical assays. Although biophysical assays did not reveal an interaction between oxo-C14-HSL and ALI1, a pull-down assay and an indirect method employing biosensor *E. coli LuxCDABE* support such interaction. We expressed fluorescently tagged ALI1 in tobacco leaves to assess the localization of ALI1 and demonstrate that ALI1 colocalizes with the plasma membrane, tonoplast, and endoplasmic reticulum.

**Conclusions:**

These results suggest that the candidate protein ALI1 is indispensable for oxo-C14-HSL-dependent priming for enhanced resistance in Arabidopsis and that the ALI1 protein may interact with oxo-C14-HSL. Furthermore, ALI1 protein is localized in the cell periphery. Our findings advance the understanding of interactions between plants and bacteria and provide an avenue to explore desired outcomes such as enhanced stress resistance, which is useful for sustainable crop protection.

**Supplementary Information:**

The online version contains supplementary material available at 10.1186/s12915-022-01464-3.

## Background

Being in constant vicinity with microorganisms, plants depend on efficient interaction and perception mechanisms between themselves and the surrounding microbiota. The canonical perception of microorganisms by eukaryotes is based on particular structural molecules or proteins. However, plants evolved also means to respond to bacterial quorum-sensing (QS) molecules. QS allows bacterial cells to communicate with each other; this process is based on the production and detection of diverse autoinducer molecules. These molecules enable bacteria to collectively coordinate behavioral changes to regulate cellular processes like biofilm production, genetic exchange, bioluminescence, motility, sporulation, and virulence, all of which are critical for survival, adaptation, and persistence in a changing environment [1, 2]. Gram-negative bacteria generally orchestrate their cellular behavior via the synthesis of *N*-acyl homoserine lactones (AHL) [3–5]. AHL molecules are comprised of two intrinsic moieties: a core *N*-acylated homoserine lactone ring and an amide linked acyl side chain ranging from 4 to 18 carbons, often with the hydrogen substituted by a hydroxyl or ketone group on the third carbon position [6–8]. Besides orchestrating the communication within bacterial populations, increasing evidence suggests that AHL elicit specific responses in the eukaryotic hosts [9]. Mathesius et al. demonstrated the first evidence of specific AHL responses in *Medicago truncatula* using a proteomic approach [10]. *N*-3-oxo-tetradecanoyl-*L*-homoserine lactone (oxo-C14-HSL) from *Ensifer meliloti* (*Sinorhizobium meliloti*) and oxo-C16:1-HSL from *Pseudomonas aeruginosa* induced auxin response and flavonoid synthesis proteins as well as secretion of small compounds that mimic QS signals that may disrupt QS mechanism in associated bacteria.

Considerable research has been conducted on the impact of different AHL on diverse plants, including physiological and transcriptional changes that influence root and shoot growth. Several studies suggested that the effects of AHL on plants are dependent on the acyl-chain length and AHL concentration. Short-chained AHL molecules, such as *N*-hexanoyl-*L*-homoserine lactone (C6-HSL), *N*-3-oxo-hexanoyl-*L*-homoserine lactone (oxo-C6-HSL), and *N*-3-oxo-octanoyl-*L*-homoserine lactone (oxo-C8-HSL), increased root length in Arabidopsis [11, 12], whereas medium-chained AHL, such as *N*-decanoyl-*L*-homoserine lactone (C10-HSL), induced lateral root and root hair formation [13]. However, the root growth-inducing effect of C6-HSL and oxo-C6-HSL was reversed when higher concentrations (> 10 μM) of these AHL were used, as inhibitory effects on root length were observed [14]. The impact of C6-HSL treatment on growth promotion was dependent on auxin signaling processes [12, 15]. AHL-induced augmentation in root length and plant biomass was also observed in wheat, barley, and cucumber [16–18]. Besides, *N*-3-oxo-decanoyl-*L*-homoserine lactone (oxo-C10-HSL) induced adventitious root formation in mung beans (*Vigna radiata*) through enhanced transport of basipetal auxin, ensuing in the accumulation of hydrogen peroxide and nitric oxide [15]. Nonetheless, some studies disputed the role of auxin in AHL-mediated modulation of the plant [13, 14].

In addition to modifying plant growth, AHL can prime plants for enhanced resistance against pathogens. Priming induces a physiological state characterized by faster and more robust defense responses when elicited by stress factors. This phenomenon strengthens the plant’s resistance and tolerance to biotic and abiotic stressors. A recent study presented oxo-C8-HSL-induced priming for enhanced resistance in Arabidopsis against *Pseudomonas syringae* pv. *tomato* (*Pst*) [19]. Former results from our group indicate that long-chain AHL oxo-C14-HSL primes plants for enhanced resistance against biotrophic and hemibiotrophic pathogens [20]. Moreover, AHL priming effects were also observed in crop plants, including barley, wheat, and tomato, attributed to oxo-C14-HSL by *E. meliloti* [21]. C6-HSL-primed wheat seeds induced a transgenerational effect of increased yield and resistance and promoted faster germination and embryo development [16]. Our recent study on complex interactions between the host plant and multiple AHL molecules containing both short-chain and long-chain AHL revealed that combinations of three or more AHL-primed plants for enhanced resistance against *Pst*, indicating enhanced resistance as a significant result of exposure to multiple AHL in Arabidopsis [22]. Furthermore, AHL-primed plants against abiotic stressors. Arabidopsis treated with oxo-C6-HSL showed increased tolerance to high salt concentrations, probably through both ABA-dependent and ABA-independent signaling pathways [23, 24]. Hydrophilic AHL treatment improved plant stress tolerance through tissue and compound-specific modification of the activity of several antioxidants and detoxifying enzymes [25]. A nanocomposite fertilizer using *N*-butanoyl-*L*-homoserine lactone (C4-HSL) coupled with magnetic carbon nanofibers induced growth, tolerance to oxidative and high salinity levels, and enhanced resistance to the fungal pathogen *Fusarium oxysporum* in chickpeas (*Cicer arientinum*) [26].

These findings suggest that plants respond specifically to different AHL molecules, implying specific perception mechanisms. Various components were identified to play a critical role in AHL-mediated responses in plants. C4-HSL-induced transient and instantaneous Ca^2+^ spiking in Arabidopsis root cells [27]. The transcription factor AtMYB44 and the Arabidopsis growth and development-related protein calmodulin (AtCaM) are required for oxo-C6-HSL-induced root elongation in Arabidopsis [28, 29]. Liu et al. suggested that the G-protein-coupled receptor GCR1 and the canonical Gα subunit GPA1 are required for oxo-C6-HSL and oxo-C8-HSL-mediated root elongation effects in Arabidopsis [30]. Different studies have verified that short-chain AHL molecules are readily taken up by roots and transported to shoots, while long-chain AHL molecules are not systemically transported, most probably due to their lipophilic nature [12, 31, 32]. Although these studies decipher plant components integral for AHL response, no studies have been conducted to decode AHL-interacting proteins in plants.

In this study, we investigated one of such components, a membrane-associated protein required for oxo-C14-HSL-mediated enhanced resistance in plants. We propose that the Arabidopsis protein *A*H*L*-Pr*i*ming Protein *1* (ALI1), previously named AtGALK2 (galactokinase 2) and currently renamed and re-annotated as AtGlcAK2 (glucuronokinase 2), is an integral component of oxo-C14-HSL-mediated AHL priming.

## Results

### Principal mechanism of AHL-induced priming is missing in ali1

To identify plant factors required for efficient AHL priming, we focused on the gene encoded by the *At5g14470* locus since this gene was identified in previous AHL-priming-related studies (data not shown). The *At5g14470* locus encodes a putative kinase from the GHMP kinase family, recently named AtGlcAK2 or glucuronokinase 2 and formerly known as AtGALK2 or galactokinase 2. However, because of unclarities in the current annotation and its primary function prediction, we propose to rename it to *A*H*L*-Pr*i*ming Protein *1* (ALI1).

In a first step, to verify if oxo-C14-HSL influences the expression of *AtGlcAK2*/*ALI1*, quantitative PCR analysis was carried out with wild-type Col-0 seedlings that were pretreated with 6 µM oxo-C14-HSL for 3 days and subsequently elicited with 100 nM flg22. The results revealed that pretreatment with oxo-C14-HSL had no impact on the expression of *At5g14470* locus encoded gene, when compared to the control (acetone)-treated Col*-*0 plants. However, 2 h after flg22 elicitation, we could observe a twofold increase in *AtGlcAK2/ALI1* expression level in the Col*-*0 plants, irrespective of the pretreatment. Furthermore, the enhanced expression of *AtGlcAK2/ALI1* in Col*-*0 due to flg22 challenge appears to be transient since the expression level returned to the basal level 24 h after flg22 challenge, irrespective of the pretreatment (Additional file 2: Fig. S1)*.* This suggests that AtGlcAK2/ALI1 might play a role in the early response to bacteria.

**Fig. 1.**
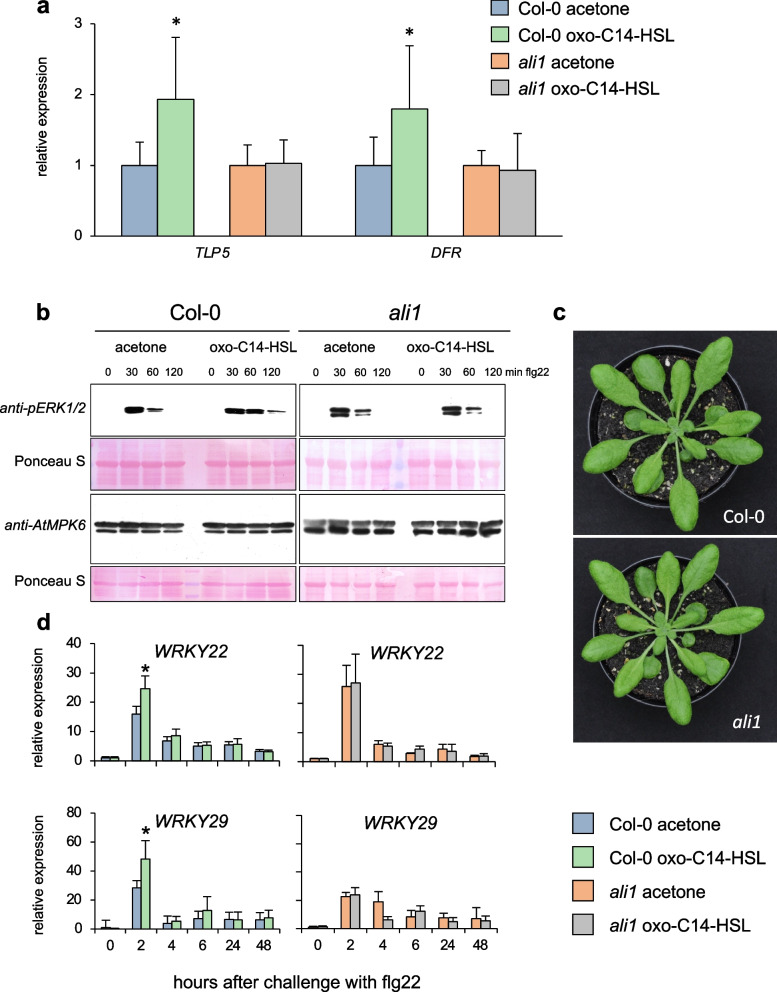
Principal mechanism of AHL-induced priming is missing in *ali1*. **a** Expression analysis of two AHL-priming marker genes (*TLP5* and *DFR*) in Col-0 and *ali1* 72 h after priming with 6 µM oxo-C14-HSL. The abundance of each gene transcript was normalized with the Ubiquitin ligase (*At5g25760*) transcript and acetone control. The bar represents mean and error bars represent SD from eight independent biological repetitions from three experimental replicates. **b** Phosphorylation pattern of the AtMPK6 in response to flg22 challenge in Col-0 and *ali1*, pretreated with 6 µM oxo-C14-HSL or acetone (control). Western blot analysis was performed in three independent experiments, representative blots are shown. **c** Representative images of Col*-*0 wild-type and the *ali1* mutant. **d** The expression profile of defense-related genes *WRKY22* and *WRKY29* was monitored at various time points after 100 nM flg22 challenge in Col-0 and *ali1* mutant. The abundance of each gene transcript was normalized with the *Ubiquitin ligase* (*At5g25760*) transcript and 0 hpt (hours post treatment) levels. Error bars represent SD from four independent biological repetitions. Experiments were carried out three times with similar results. In **a** and **d**, data are presented as mean ± SD. * *P* ≤ 0.05 (Student’s *t* test), individual data values for **a** and **d** in Additional File 1

To further corroborate the role of AtGlcAK2/ALI1 during AHL priming, we used a mutant line with a T-DNA insertion in *At5g14470* locus, along with wild-type Col-0. The segregating line N560407 was obtained from Salk Institute and propagated, and a non-segregating line (*ali1*) was characterized in a PCR approach (Additional file 2: Fig. S2). We performed quantitative PCR and western blot-based kinase assays to verify whether AHL priming depends on the presence of functional AtGlcAK2/ALI1 in Arabidopsis [20]. Higher expression of *TLP5* and *DFR* genes, usually observed upon AHL priming [33], was missing in the *ali1* mutant (Fig. 1a). Furthermore, both Col-0 and the *ali1* mutant responded to the flg22 challenge and activated MPK6 transiently. In control Col-0 plants, the phosphorylation on the MPK6 activation loop was detectable 30 min after challenge with 100 nM flg22, but the phosphorylation level declined 60 min post-challenge. No phosphorylation of the activation loop was observed 120 min after the flg22 challenge. On the contrary, oxo-C14-HSL-pretreated plants exhibited enhanced and prolonged activation of MPK6 since the pTEpY epitope was observed until 120 min after the flg22 challenge (Fig. 1b and Additional file 2: Table S1). In *ali1* mutant pretreated with either acetone or oxo-C14-HSL, the pattern of MPK6 phosphorylation was identical to control Col-0 plants (Fig. 1b and Additional file 2: Table S1). These results indicate that the AtGlcAK2/ALI1 is required for the oxo-C14-HSL-mediated enhanced and prolonged activation of MPK6 in response to the flg22 challenge in AHL-primed plants.

**Fig. 2.**
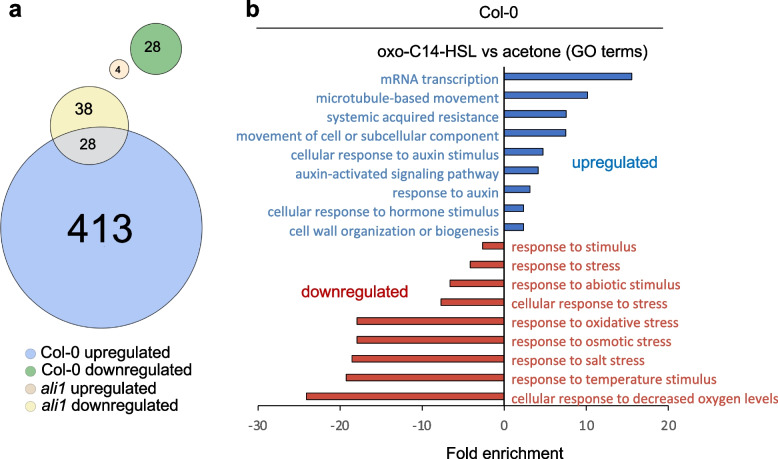
AHL priming causes differential expression of only few genes in *ali1* mutant. **a** Euler diagram of differentially expressed genes after pretreatment with 6 µM oxo-C14-HSL and challenge with 100 nM flg22 for 2 h. The upregulated and downregulated genes from Col-0 are shown in blue and green, respectively, whereas the upregulated and downregulated genes from *ali1* are shown in light brown and yellow, respectively*.* **b** The bar plot indicates significantly enriched GO terms of *Arabidopsis* genes differentially expressed upon oxo-C14-HSL pretreatment and following flg22 challenge, upregulated are shown in blue and downregulated genes in red. In **a**, differentially expressed genes were calculated based on *q*-value < 0.05 and fold change > 2 from three independent replicates

To determine whether AHL priming reflected by enhanced upregulation of defense-related genes after a challenge [20, 34, 35] is influenced by the absence of *ALI1*, we assessed genes’ expression using a qPCR approach. Analysis of *WRKY22* and *WRKY29* expression in Col-0 and *ali1* revealed that the seedlings could perceive 100 nM flg22 and elicit a response (Fig. 1d). However, the comparison between Col-0 and *ali1* revealed higher transcript abundance in oxo-C14-HSL-pretreated Col-0 seedlings. This difference was missing in the *ali1* mutant. Additional expression profiles of *GST6* and *Hsp70* revealed similar results (Additional file 2: Fig. S3). To comprehensively investigate whether the *ali1* mutant responds to oxo-C14-HSL, we carried out an additional whole transcriptome analysis. Using an RNA sequencing approach, we examined differences in gene expression of plants challenged with 100 nM flg22 after priming with 6 µM oxo-C14-HSL using an RNA sequencing approach. Differentially expressed genes were set as showing a log_2_ fold change of 1 or more and a moderate *p* < 0.05 (Additional file 3, 4, 5 and 6: Datasets S1-S4). Comparison between control and oxo-C14-HSL-pretreated Col-0 seedlings challenged with 100 nM flg22 revealed upregulation of 441 (Additional file 3: Dataset S1) and downregulation of 28 genes (Additional file 4: Dataset S2). A similar comparison between control and oxo-C14-HSL-pretreated *ali1* revealed upregulation of four (Additional file 5: Dataset S3) and downregulation of 66 genes (Additional file 6: Dataset S4) (Fig. 2). Gene Ontology (GO) analysis of Col-0 genes differentially expressed between oxo-C14-HSL-pretreated and control plants, 2 h after the challenge with flg22 showed enrichment of several terms. These are associated with mRNA transcription, movement of cellular components, systemic acquired resistance, cell wall organization, auxin-activated signaling pathway, and cellular response (Fig. 2b). The differentially downregulated genes showed enrichment for GO terms associated with abiotic stimulus related to decreased oxygen levels, temperature, salt, osmotic and oxidative stress in the case of Col-0 (Fig. 2b), and glucosinolate metabolism in the case of *ali1*. Taken together, the upregulation of defense-related genes, a typical phenomenon in AHL-primed plants, seems to depend on the presence of AtGlcAK2/ALI1.

**Fig. 3.**
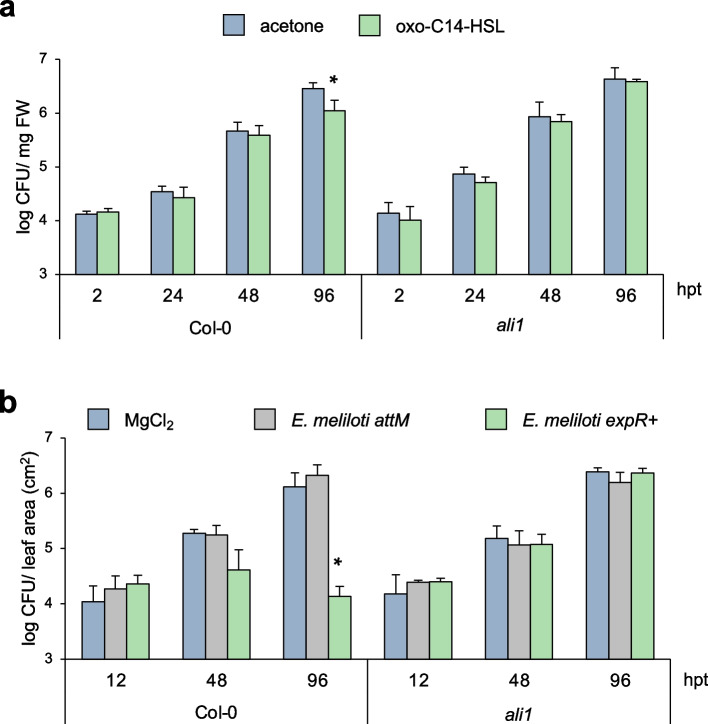
Oxo-C14-HSL-induced resistance in Arabidopsis against *Pst* requires the presence of ALI1. **a** Pathogenicity assays with *Pseudomonas syringae* pathovar *tomato* (*Pst*) on wild-type Col*-*0 and *ali1* mutant plants grown for 3 weeks on ½-strength MS containing acetone or 6 µM oxo-C14-HSL and then challenged with *Pst.* The bar represents the mean and error bar represents SD of three replicates. Experiments were performed three times with similar results. **b** Pathogenicity assays with *Pst* on wild-type Col-0 and *ali1* mutant grown on soil, pretreated three times with 10 mM MgCl_2_ (control), *Ensifer meliloti attM* or *E*. *meliloti expR* + and then infiltrated with *Pst*. The bar represents the mean and error bar represents SD of four replicates. Experiments were carried out three times with similar results. In **a** and **b**, data are presented as mean ± SD. * *P* ≤ 0.05 (Student’s *t* test), individual data values for **a** and **b** in Additional File 1

To assess whether the absence of the *AtGlcAK2*/*ALI1* gene impacts on the oxo-C14-HSL-priming for enhanced resistance, we performed two different *Pseudomonas syringae* pathovar *tomato* (*Pst*)-based pathogen assays. The first assay was performed using the AHL molecule under sterile conditions. *Pst* proliferated on *Arabidopsis* leaves from c. 10^4^ colony-forming units (CFU) mg^−1^ FW leaf (2 h after inoculation) to about 10^7^ CFU mg^−1^ FW leaf (96 h after the inoculation) (Fig. 3a). However, we observed a reduction in the bacterial CFU number, 96 h after *Pst* inoculation in Col-0 seedlings that were grown on oxo-C14-HSL-supplemented media when compared to control plants. In contrast to the wild type, no enhanced resistance was observed in the *ali1* mutant (Fig. 3a). We performed a similar assay using the oxo-C14-HSL-producing bacterium *Ensifer meliloti expR* + [21]. In a glasshouse experiment, plants were inoculated three times over 3 weeks with either 10 mM MgCl_2_ (control), the *E.* *meliloti attM* strain unable to accumulate the oxo-C14-HSL molecule (bacterial control), or the *E.* *meliloti expR* + strain. Leaves of Col*-*0 and *ali1* were infiltrated with *Pst* suspension of 2 × 10^7^ CFU ml^−1^ and the presence of *Pst* was monitored for 96 h. A significant reduction of the bacterial count was observed only in leaves of Col*-*0 plants, pretreated with the *E. meliloti expR* + strain (Fig. 3b). This indicates that the AHL priming for enhanced resistance with oxo-C14-HSL is lost in plants when the *AtGlcAK2/ALI1* gene is nonfunctional.

### YFP-ALI1 is associated with the plasma membrane, tonoplast, and endoplasmic reticulum

**Fig. 4.**
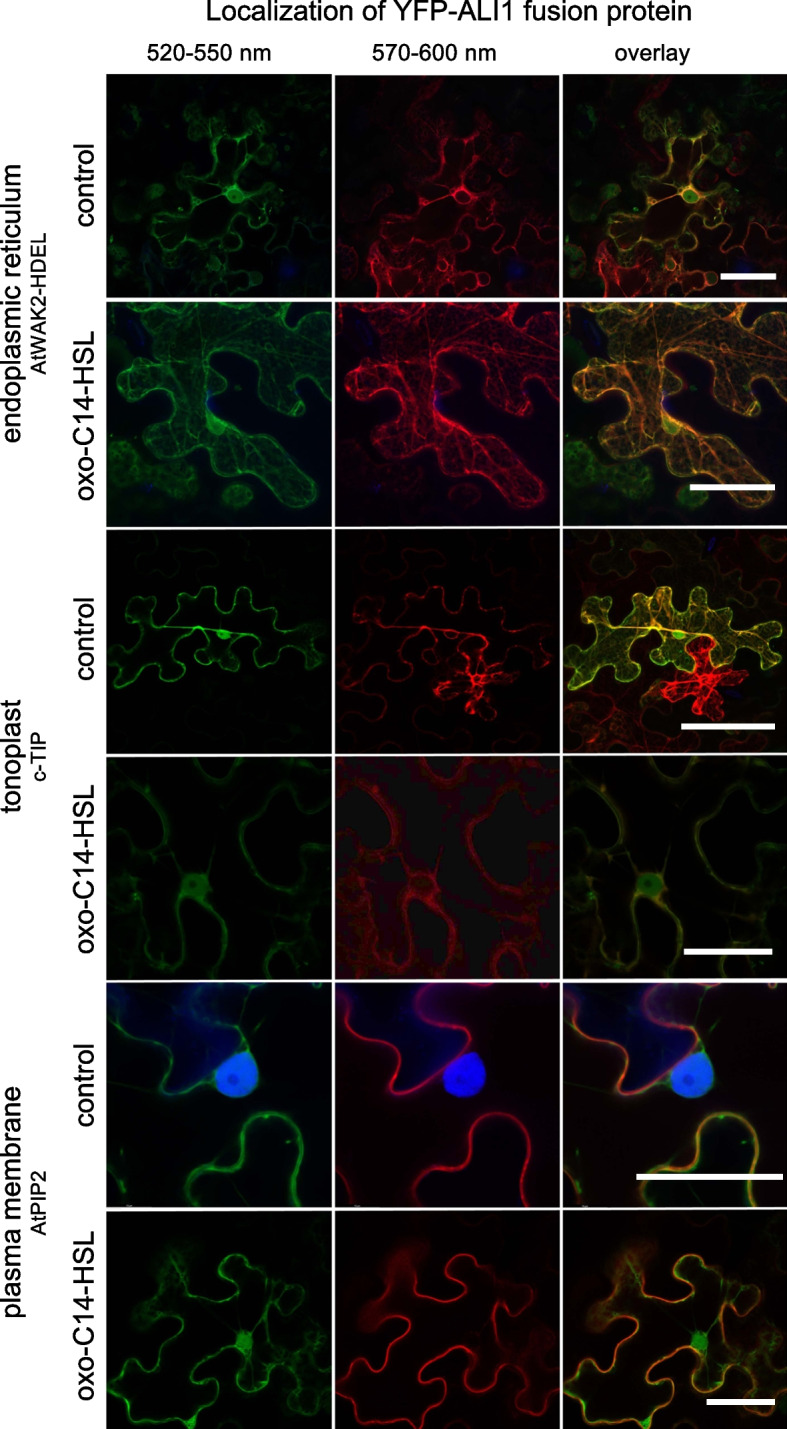
ALI1 protein is localized at the cell’s periphery in PM, ER, and tonoplast and does not translocate after oxo-C14-HSL treatment. Confocal images displaying the subcellular localization of YFP-tagged ALI1 before and after oxo-C14-HSL treatment when transiently expressed in cells of *N.* *benthamiana* leaves. Plasmids carrying YFP-tagged ALI1 version and mCherry-marked proteins localizing in ER (AtWAK2-HEDL), tonoplast (c-TIP), or plasma membrane (AtPIP2) were co-transformed to *N. benthamiana* leaf epidermal cells by *Agrobacterium*-infiltration, and further re-infiltrated after 24 h at the same co-transformed spots of a leaf with 6 µM oxo-C14-HSL. The left panel shows in green, fluorescence of YFP-tagged ALI1, whereas the middle panel shows in red, fluorescence of differently localized mCherry-marked proteins (Additional file 2: Table S3). The right panel shows corresponding merged images, and yellow color indicates colocalization. Scale bar = 40 μm

Thereupon, we examined the cellular localization of AtGlcAK2/ALI1. To this end, leaves of 4-week-old tobacco (*Nicotiana benthamiana*) were infiltrated with a mixture *of Agrobacterium tumefaciens* GV3101 carrying a plasmid with sequences of *YFP*-tagged *AtGlcAK2/ALI1* and LBA4404 carrying a plasmid with mCherry-tagged gene sequences of proteins that localizes at diverse subcellular locations (Additional file 2: Table S3) [36]. The results revealed that the YFP-ALI1 fusion protein colocalized with the plasma membrane, endoplasmic reticulum (ER), and partially with the tonoplast (Fig. 4**)** and not with other subcellular organelles including Golgi, nucleolus, plastids, and peroxisome (Additional file 2: Fig. S4) and cell wall as indicated after the plasmolysis (Additional file 2: Fig. S5). Interestingly, we observed no translocation of the YFP-ALI1 fusion protein after an oxo-C14-HSL treatment (Fig. 4).

### ALI1 has a strong affinity to the bacterial quorum-sensing molecule, oxo-C14-HSL

**Fig. 5.**
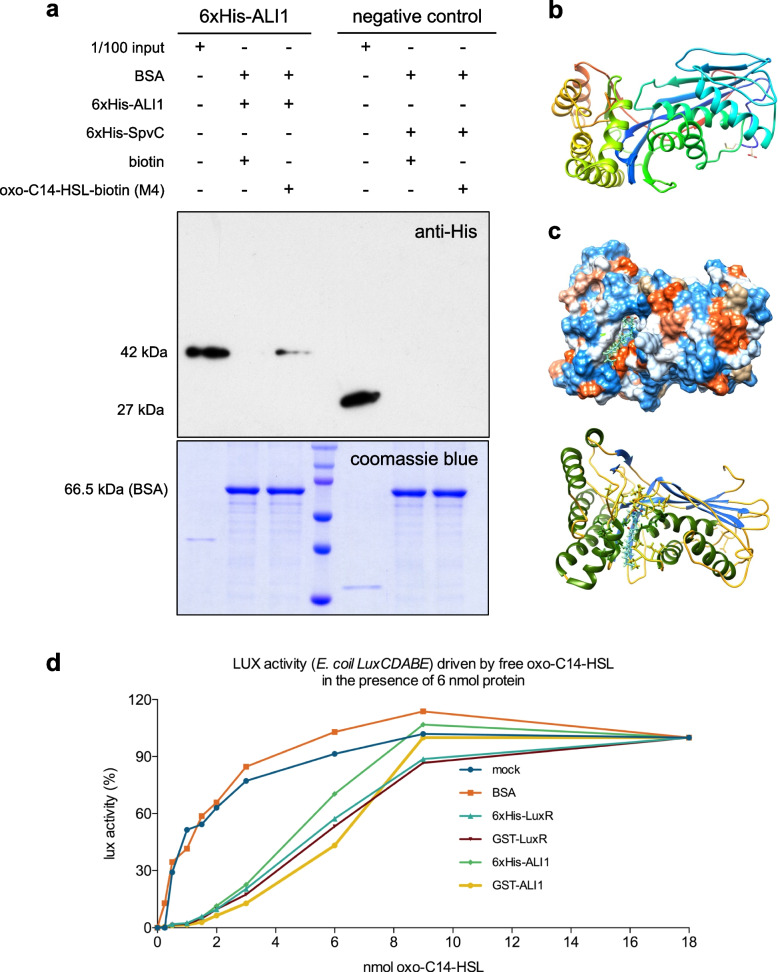
ALI1 interacts with oxo-C14-HSL. **a** Pull-down analysis between the His-tagged ALI1 and oxo-C14-HSL (M4) and detection of retained ALI1 on beads bound to M4 but not on beads bound to biotin. The upper panel presents a western blot, in which either 1/100 of the input proteins (6xHis-ALI1 or 6xHis-SpvC, as negative control) or proteins resulting from a pull-down were separated, blotted, and probed with anti-His antibody. The pull-down setup is indicated above the panel. Pull-down analysis was performed in three independent experiments. A representative blot from those independent experiments is shown. The lower panel presents loading control for the pull-down setup, and input shows 1/100 of the protein amount used for the pull-down. **b** The tertiary structure of ALI1 protein. (Predicted structure of ALI1 with threading or fold recognition model after docking simulations with oxo-C14-HSL). **c** Protein threading or fold recognition predicted model of ALI1 with docked oxo-C14-HSL ligand, simulated in the online docking webserver SwissDock. **d** Luminescence activity assay indicating the binding of oxo-C14-HSL to ALI1. The binding capacity was assessed by determining the concentration of free oxo-C14-HSL using the *E. coil LuxCDABE* reporter strain after an overnight incubation with 6 nmol ALI1 or LuxR (positive control) and BSA (negative control) with different amounts of oxo-C14-HSL, as indicated. The individual data values for **d** in Additional File 1

In order to assess if bacterial quorum-sensing molecules interact with plant protein(s), we took advantage of the biotinylated derivative of the *N*-3-oxo-tetradecanoyl-*L*-homoserine lactone (oxo-C14-HSL) molecule, M4 [37]. We sought to verify the interaction between oxo-C14-HSL and AtGlcAK2/ALI1 using the fusion 6xHis-ALI1 protein and M4. 6xHis-ALI1 protein was retained on beads bound to M4 but not on beads exposed to the biotin control, suggesting positive interaction between the ALI1 and M4 (Fig. 5a). A 6xHis-tagged Salmonella Plasmid Virulence C (SpvC) protein, a virulence protein in *Salmonella enterica*, was used as a negative control to verify that M4 does not bind to the 6xHis-tag. Modeling the predicted interaction between AtGlcAK2/ALI1 and oxo-C14-HSL revealed that the ligand could interact with the central groove of the protein (Fig. 5b,c and Additional file 2: Fig. S6).

To further assess the potential interaction between AtGlcAK2/ALI1 and oxo-C14-HSL, we performed a microscale thermophoresis (MST) assay. The combined dose–response curve from both experimental replicates indicated no interaction between ALI1 and oxo-C14-HSL in the analyzed ligand concentration range and under the applied experimental conditions (Additional file 2: Fig. S7a). One of the reasons for the low chances of observing a binding event could be the distant location of the dye in respect to the protein–ligand binding site. Therefore, we also examined the influence of oxo-C14-HSL on the thermal stability of AtGlcAK2/ALI1 protein by characterizing its thermal stability and unfolding profile utilizing a nano-differential scanning fluorimetry (nanoDSF) assay. For 6xHis-ALI1, two clear unfolding transitions at 50.88 °C (inflection point #1) and 66.51 °C (inflection point # 2) were observed while for 6xHis-SpvC, one clear unfolding transition at 43.99 °C (inflection point #1) was observed (Additional file 2: Fig. S7b). The presence of oxo-C14-HSL led to a shift of − 1.91 °C (∆ IP #1 column) and − 0.88 °C (∆ IP #2 column) in 6xHis-ALI1 and − 0.80 °C (∆ IP #1 column) in 6xHis-SpvC (Additional file 2: Fig. S7b). Since both proteins (6xHis-ALI1 and 6xHis-SpvC) showed comparable thermal shifts, we concluded that this was probably an artifact. However, thermal shift assays can be biased by unspecific effects such as a change in ionic strength, impurities that increase with increasing ligand concentration, or other unspecific binding events. Both proteins were shown to be destabilized by a ligand molecule implying that interaction between ALI1 and oxo-C14-HSL was not observed under the applied experimental conditions, which led us to an indirect method.

The concentration of free oxo-C14-HSL was monitored in the presence of both 6 nmol 6xHis-ALI1 or 6 nmol 6xHis-LuxR (used as a positive control) and BSA (used as a negative control). Proteins were incubated overnight with different concentrations of oxo-C14-HSL, and the presence of free molecules was monitored using the reporter *E. coli* strain (*E. coli LuxCDABE*). As revealed, mock and BSA variants induced about 80 to 100% of the possible lux activity (light production capacity) in the reporter *E. coli* strain in the presence of 3 nmol oxo-C14-HSL, indicating that the molecule is freely available. Moreover, 3 nmol oxo-C14-HSL seemed to be sufficient to fully induce the *LuxCDABE* operon. In comparison, the presence of 6 nmol 6xHis-LuxR from *E. meliloti* or 6 nmol of 6xHis-ALI1 from *Arabidopsis* in the system inhibited the induction of the *LuxCDABE* operon significantly. This inhibition was observable until the 9 nmol oxo-C14-HSL variant. In the presence of 6 nmol oxo-C14-HSL (equimolar amount), 50% of the possible *LuxCDABE*-originated luminescence was observed. We concluded, therefore, that LuxR (the native oxo-C14-HSL receptor from *E. meliloti*) and AtGlcAK2/ALI1 are equivalently able to sequestrate oxo-C14-HSL molecule from the solution (Fig. 5d).

### AHL-induced priming is restored in complemented ali1 and ALI1-dependent phenotypes are oxo-C14-HSL specific

**Fig. 6.**
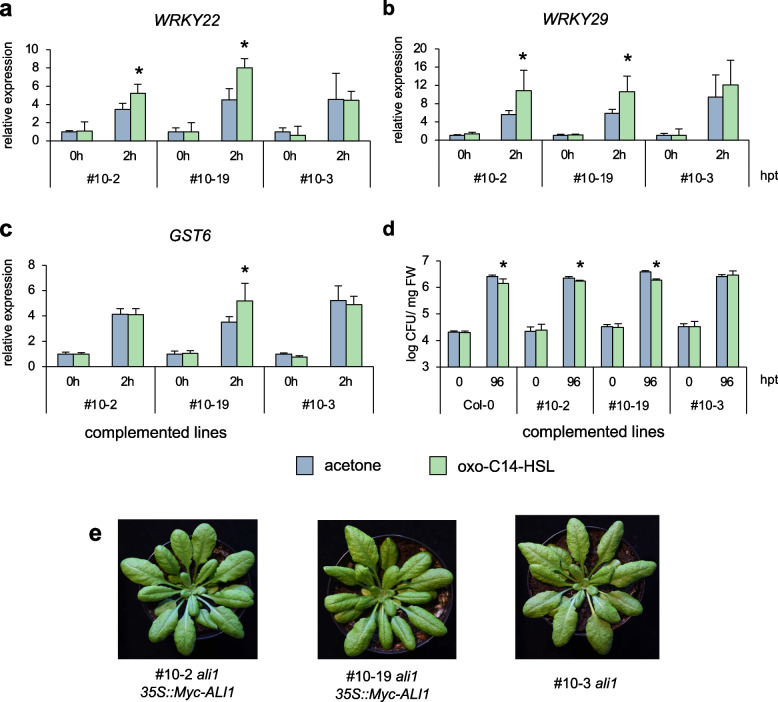
AHL-induced priming is restored in complemented *ali1*. The expression profile of three defense-related genes **a** *WRKY22*, **b** *WRKY29*, and **c** *GST6* were monitored before (0 h) and after (2 h) 100 nM flg22 challenge in two complemented lines of *ali1:* #10–2 and #10–19 as well as an outcross (non-complemented) line #10–3 that were earlier primed with 6 µM oxo-C14-HSL for 3 days. The abundance of each gene transcript was normalized with the Ubiquitin ligase (*At5g25760*) transcript and acetone control at 0 hpt before flg22 elicitation. The bar represents the mean and error bar represents SD of three replicates. Experiments were carried out two times with similar results. **d** Pathogenicity assays with *Pst* on wild-type Col*-*0, two complemented lines of *ali1:* #10–2 and #10–19 as well as an outcross (non-complemented) line #10–3. Plants grown for 3 weeks on ½-strength MS containing acetone or 6 µM oxo-C14-HSL and then challenged with *Pst.* The bar represents the mean and error bar represents SD of three replicates. Experiments were carried out two times with similar results. **e** Representative photographs of 6-week-old #10–2, #10–19, and #10–3 plants. In **a–d**, data are presented as mean ± SD. * *P* ≤ 0.05 (Student’s *t* test), individual data values for **a–d** in Additional File 1

In order to assess if the reintroduction of the *AtGlcAK2/ALI1* gene restores oxo-C14-HSL-dependent phenotypes, we performed gene expression analysis and *Pst* assay using homozygous *ali1* lines expressing Myc-tagged ALI1 under the *35S* promoter (*35S::10xMyc-ALI1*); line #10–2 and #10–19 together with an outcrossed line #10–3 (used as a negative control) (Additional file 2: Fig. S8). The expression patterns in both complemented #10–2 and #10–19 and the outcrossed (non-complemented) line #10–3 revealed that all seedlings responded to the flg22 challenge (Fig. 6a–c). Furthermore, expression levels of defense-related *WRKY22*, *WRKY29*, and *GST6* genes 2 h after flg22 challenge in the complemented #10–19 line, and *WRKY22*, *WRKY29* in the #10–2 line showed higher transcript abundance when pretreated with oxo-C14-HSL (Fig. 6a–c). This difference was missing in the outcrossed line #10–3. Similarly, the *Pst* assay validated that reintroducing *AtGlcAK2/ALI1* gene into the *ali1* mutant reinstates the ability of AHL priming. We observed a reduction in the proliferation of *Pst* in oxo-C14-HSL-treated plants compared to control plants in Col-0, 96 h after inoculation (Fig. 6d). Furthermore, we observed a similar reduction in *Pst* proliferation in complemented lines, #10–2 and #10–19 after an oxo-C14-HSL pretreatment, indicating enhanced resistance due to AHL priming (Fig. 6d). This reduction was missing in the non-complemented line #10–3 since no difference in the CFU count between the treatments was observed (Fig. 6d). These results suggest that reintroducing *AtGlcAK2/ALI1* into the *ali1* background restores the ability of AHL priming.

**Fig. 7.**
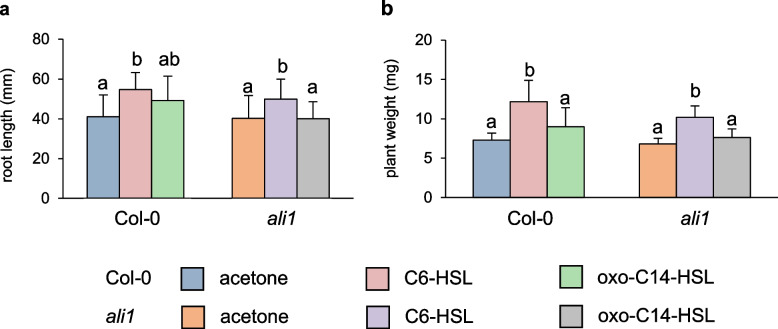
ALI1-dependent phenotypes are oxo-C14-HSL specific. Analysis of **a** root growth and **b** plant weight of Col-0 and *ali1* plants grown for 4 weeks on ½-strength MS containing acetone, 6 µM C6-HSL or 6 µM oxo-C14-HSL (*n* = 10 plants for each group). Data are presented as mean ± SD, and different letters indicate *P* ≤ 0.05 (Tukey’s HSD post hoc test). Experiments were carried out three times with similar results, individual data values for **a** and **b** in Additional File 1

Finally, we aimed to investigate if *ALI1-*dependent phenotypes are specific to oxo-C14-HSL. Our previous reports described AHL-induced growth promotion in plants treated with a short-chained AHL molecule, C6-HSL [20, 22]. We performed, therefore, a root growth assay on 1-week-old Col-0 and *ali1* seedlings on ½-strength MS agar supplemented with either 6 µM oxo-C14-HSL or 6 µM C6-HSL. The plants were grown vertically to observe the root growth for 4 weeks. We observed that C6-HSL treatment enhanced root length in Col-0 (Fig. 7a). Moreover, we observed a similar phenomenon of longer roots upon C6-HSL treatment in *ali1* seedlings (Fig. 7a). There was no significant difference in the root length of both Col-0 and *ali1* seedlings between control and oxo-C14-HSL treatments (Fig. 7a). Similarly, we also observed that both Col-0 and *ali1* seedlings growing on plates containing 6 µM C6-HSL had higher biomass (Fig. 7b). Taken together, the influence of short-chained AHL molecules on plant growth does not seem to depend on the presence of functional AtGlcAK2/ALI1.

## Discussion

In this study, we presented several levels of evidence that ALI1 encoded by the *At5g14470* locus is required for oxo-C14-HSL-induced priming for enhanced resistance. ALI1 is a putative protein kinase belonging to the GHMP (galactose, homoserine kinase, mevalonate kinase, phosphomevalonate kinase) super kinase family protein. This protein was formerly annotated as galactokinase 2 (AtGALK2) and was proposed to catalyze the phosphorylation of α-D-galactose (Gal). However, the only galactokinase in Arabidopsis is encoded by another locus *At3g06580* [38]. Here, we propose that the currently annotated as glucuronokinase 2 (AtGlcAK2) protein, supposedly an isoform of glucuronokinase 1 (AtGlcAK1), which catalyzes the phosphorylation of D-glucuronic acid, should be renamed to ALI1. Pieslinger et al. demonstrated that amino acid sequences of both proteins encoded by the *At3g01640* locus (AtGlcAK1) and the *At5g14470* locus are highly homologous, predicting the latter gene to be the second isoform of glucuronokinase, AtGlcAK2 [39]. Another study on AtGlcAK1 by the same group suggested that the ineffective loss of function of *glcak1* mutants was probably due to the activity of the other isoform, AtGlcAK2 [40]. However, enzymatic analysis has yet to confirm the protein function of AtGlcAK2. Despite being highly homologous to the AtGlcAK1, there is no evidence that AtGlcAK2 has glucuronokinase activity of catalyzing the phosphorylation of D-glucuronic acid. According to another study, the gene encoded by *At5g14470*, which was erroneously designated as AtGALK2 in this study, is involved in root and flower development, the abscisic acid signaling pathway, and stress response influencing the expression of various ABA-related genes [41]. Nonetheless, we propose a new function for this protein based on our experimental findings.

Previously, Thomanek et al. demonstrated that the biotin-tagged oxo-C14-HSL probe directly interacts with the bacterial LuxR-type receptor. Here, we used the biotin-tagged probe for a pull-down assay with Arabidopsis proteins [37]. Additional pull-down assay with a His-tagged ALI1 revealed that ALI1 indeed might interact with oxo-C14-HSL. Other binding assays, such as the MST and nanoDSF assays, could not confirm the interaction. However, it does not rule out the possibility of interaction since we could ensure an oxo-C14-HSL-sequestering ability of AtGlcAK2/ALI1 using an indirect technique based on the detection of free oxo-C14-HSL by the bacterial biosensor *E. coli LuxCDABE*.

MAPKs translate extracellular signal perception into intracellular responses [42, 43]. They are also linked to priming in plants, as AHL priming for enhanced resistance resulted in robust and extended activation of MAP kinases in Arabidopsis and barley [20, 34, 35, 44]. Furthermore, several genes, including the WRKY transcription factors *WRKY22* and *WRKY29*, *GST6*, and *Hsp70*, are upregulated during oxo-C14-HSL-priming in Arabidopsis [22, 33, 35]. Our current findings are consistent with these studies, as oxo-C14-HSL-primed wild-type Col-0 showed stronger and prolonged activation of MAP kinases and increased expression of mentioned genes. The *ali1* mutant showed neither increased kinase activity nor an augmented expression of defense-related genes, both hallmarks of AHL priming. The enhanced resistance against pathogens in Arabidopsis and other crop plants, including barley and tomato, induced by the *Ensifer meliloti* was attributed to oxo-C14-HSL produced by *E. meliloti* [21, 35, 45]. Similarly, the pathogenicity assay with *Pseudomonas syringae* pv. *tomato* (*Pst*) indicated that the AHL priming for enhanced resistance is absent in plants lacking functional *ALI1*. Nonetheless, AHL priming is restored by reintroducing the *ALI1* gene into the *ali1* mutant, as we observed enhanced resistance against *Pst* in complemented plants after AHL priming but not in outcrossed lines. Therefore, we concluded that the presence of functional ALI1 is required for oxo-C14-HSL-induced priming for enhanced resistance.

A previous study from our group found that oxo-C14-HSL-priming increased the expression of genes involved in Ca^2+^ signaling, G-proteins, defense, cell wall, and flavonoid metabolism using transcriptomic approaches [33]. Furthermore, in response to flg22, oxo-C14-HSL-primed Col-0 showed upregulation of genes associated with systemic acquired resistance, cell wall organization, auxin-activated signaling pathway, and movement of cellular components while downregulation of genes related to abiotic stimulus such as temperature, salt, decreased oxygen levels, and osmotic and oxidative stress. In *ali1* mutants, however, none of those GO terms were regulated, while genes involved in glucosinolate metabolism were downregulated. Overall, the upregulation of defense-related genes, a typical phenomenon in AHL-primed plants, appears to depend on the presence of ALI1.

YFP-ALI1 colocalizes with the endoplasmic reticulum (ER), tonoplast, and the plasma membrane (PM). PM and ER, in particular, contain components essential for signal transduction and homeostasis regulation. ER-PM contact sites (EPCS) are functional signaling hubs formed when ER anchors and couples with PM [46]. Since there was no translocation of ALI1 observed after an oxo-C14-HSL treatment, signal transduction is most likely mediated by the activity of ALI1 or its interacting proteins rather than translocation. Although the actual downstream events are unknown, several components have already been proposed for other AHL molecules. A previous study found that in Arabidopsis, oxo-C6-HSL and oxo-C8-HSL induce root elongation by activating the G-protein-coupled receptors GCR1 and GPA1 [30]. Furthermore, the transcription factor AtMYB44 and the Arabidopsis growth and development-related protein calmodulin (AtCaM) are involved in oxo-C6-HSL-induced root elongation in Arabidopsis [28, 29]. Those specific components are, however, most likely involved in response to a particular, in this case, short-chained AHL molecule(s) and are not required for ALI1-mediated responses to oxo-C14-HSL.

## Conclusions

We presented here evidence from several levels that AtGlcAK2/ALI1 is required for the oxo-C14-HSL-induced primed state in Arabidopsis. Different physiological assays used in this study with *ali1* mutant show that the plant responses to flg22 elicitor are similar to naïve plants despite oxo-C14-HSL treatment. A plant’s ability to be primed with oxo-C14-HSL is entirely lost in the absence of a functional *ALI1* gene as only AHL-primed wild-type Arabidopsis exhibited enhanced resistance against *Pst,* while the mutant did not. However, this phenomenon was reverted once the candidate gene was reintroduced into the mutant, as was shown by the expression of priming marker genes and pathogenic assays with *Pst*. The current study shows that the candidate protein ALI1 might interact with oxo-C14-HSL. Although biophysical assays did not demonstrate the interaction between oxo-C14-HSL and ALI1, pull-down assay and indirect technique using biosensor *E. coli LuxCDABE* successfully substantiated that the candidate protein ALI1 might interact with oxo-C14-HSL. Transcriptomic analysis showed that flg22 elicitation of AHL-primed wild-type Col-0 plants resulted in enhanced expression of genes involved in systemic acquired resistance, cell wall organization, and auxin-activated signaling pathway while downregulated genes were related to the abiotic stimulus. However, AHL pretreatment did not have any impact on the *ali1* mutant. Furthermore, assessing the localization of the candidate protein in plants showed that the ALI1 proteins are localized in the ER, PM, and tonoplast. This study will enable identifying AHL priming mechanisms and different plant factors that mediate AHL priming after AHL perception, which could be exploited through plant breeding and novel genetic technologies to improve AHL priming effects in plants. From a larger perspective, this research will help us uncover another layer of coevolutionary inter-kingdom communication between plants and bacteria, which involves quorum sensing molecules that confer plant benefits by priming plants for enhanced resistance.

## Methods

### Plant materials and growth conditions

Seeds from wild-type *Arabidopsis thaliana* Col*-*0 and the *ali1* (N560407; *At5g14470*) mutant obtained from the Salk Institute, as well as complemented (pGWB21; *35S::10xMyc-ALI1*) and outcrossed lines, were used in the study. Seed sterilization and Arabidopsis growth were carried out as was described in Shrestha et al. [22]. For the MAP kinase assay, gene expression analysis, and RNA sequencing approaches, 2-week-old seedlings were transferred to six-well plates with 3 ml ½-strength MS medium prior to pretreatment with *N*-acyl homoserine lactone (AHL). Root growth assay and sterile *Pseudomonas syringae* pathogenicity assay were performed as described in Shrestha et al. [22], except for AHL molecules used. For non-sterile *P. syringae* pathogenicity assay and *ali1* mutant complementation, 2-week-old seedlings were transferred to pots with standard bedding soil (Fruhstorfer Erde: Perlite (1:1)) and grown under short-day conditions at 8 h:16 h (light: dark) photoperiod at 22 °C for another 3 weeks in the glasshouse. Six-week-old plants were grown under long-day conditions (light:dark 16 h:8 h and 22 °C photoperiod, light intensity of 150 µmol m^−2^ s^−1^ and 60% humidity) to induce flowering.

### Characterization of ali1 mutant

For genotyping of the T-DNA insertion *ali1* mutants, PCR-based screening was performed for knockout mutation*.* Genomic DNA was extracted from wild type Col-0, mutant *ali1* and T-DNA insertion mutant that were growing on soil for 3 weeks using Qiagen DNeasy Plant Mini Kit following the manufacturer’s protocol. The first PCR was performed with left border primer LBb1-3 and gene-specific forward primer (Additional file 2: Table S1). The band was observed only in *ali1* mutant and not in the other T-DNA insertion mutant and wild-type Col-0 indicating that T-DNA was indeed inserted in *ALI1* locus (Additional file 2: Fig. S2). Similarly, the second PCR that was performed with *ALI1* gene-specific primers (Additional file 2: Table S1) resulted in the band in wild-type Col-0 and T-DNA insertion mutant but not in *ali1* mutant validating that there is a modification in the gene (Additional file 2: Fig. S2). Furthermore, third PCR was carried out with *ALI1* cDNA-specific primers (Additional file 2: Table S1) to verify whether the transcription of *ALI1* gene was impeded or not. The band was seen in cDNA of Col-0 pretreated with both oxo-C14-HSL and acetone control but not for *ali1*.

### Pretreatment with AHL

Pretreatment with AHL was conducted as in Shrestha et al. [22], except for the AHL molecules used. *N*-hexanoyl-*L*-homoserine lactone (C6-HSL) and *N*-3-oxo-tetradecanoyl-*L*-homoserine lactone (oxo-C14-HSL) (Sigma-Aldrich) at concentrations as indicated were used in this study.

### MAP kinase activity assay

Arabidopsis seedlings Col*-*0 and *ali1* (N560407) mutant were pretreated with 6 µM oxo-C14-HSL or acetone for 3 days and collected 0, 30, 60, or 120 min after eliciting with 100 nM flg22 peptide. Protein extraction from Arabidopsis seedlings and western blotting were performed as in Shrestha et al. [35].

### Gene expression analysis

Wild-type Col*-*0 and *ali1* (N560407) Arabidopsis seedlings pretreated with oxo-C14-HSL were harvested at 0, 2, 4, 6, 24, and 48 h post 100 nM flg22 treatment. Extraction of RNA and synthesis of cDNA was carried out as described in Shrestha et al. [22]. Quantitative RT-PCR (qPCR) was performed using primers listed in Additional file 2: Table S2 [20, 47]. All expression levels were normalized to the expression of *Ubiquitin ligase* (*At5g25760*).

### cDNA library construction, sequence processing, and transcriptome analysis

We performed transcriptome analysis of Col-0 and *ali1* seedlings that were pretreated for 3 days with 6 µM oxo-C14-HSL or acetone control and subsequently elicited with 100 nM flg22 in order to determine the differences in their defense responses. All treatments were performed in triplicates. RNA was isolated from Arabidopsis seedlings using RNeasy Plant Mini Kit (Qiagen) according to the manufacturer’s recommendations. One microgram of total RNA was taken for DNAse digestion using the PerfeCTa DNAse I (Quanta Biosciences), and subsequently, cDNA synthesis was carried out using the qScript cDNA Synthesis kit (Quanta Biosciences) according to the manufacturer’s recommendations. Library construction and sequencing were performed on BGISEQ-500 (BGI Tech Solutions, Hong Kong). Raw sequencing reads were cleaned by removing adaptor sequences, reads containing poly N-sequences and low-quality reads. The cleaned sequence reads (100 bp single end) were analyzed using RNAStar (Version 2.4.0d-2) [48], cufflinks (Version 2.2.1.0), cuffmerge (Version 2.2.1.0), and cuffdiff (Version 2.2.1.5) [49]. The FPKM (fragments per kilobase million) and the significant differences between transcriptional profiles of Arabidopsis seedlings Col-0 and *ali1* between the treatments were calculated based on *q*-value < 0.05 and fold change > 2 (Additional file 3, 4, 5 and 6: Dataset S1-S4). Significantly enriched genes related to specific GOs (Gene Ontology) were identified using web-based tool PANTHER (http://geneontology.org) using the PANTHER Overrepresentation Test (Released 2020–02-14), *Arabidopsis thaliana* (TAIR), GO biological process complete, and Fisher’s exact test with false discovery rate (FDR). The raw data were uploaded to GEO NCBI Sequence Read Archive with the number GSE156726. The Euler diagram was created using the R package “eulerr” [50].

### Inoculation with Ensifer meliloti strains

*Ensifer meliloti* Rm2011 *expR* + (M. McIntosh) and *E. meliloti* Rm2011 (pBBR2-attM) carrying the lactonase gene *attM* from *Agrobacterium tumefaciens* [45] were grown in Tryptone Yeast extract (TY) medium until the OD_600_ reached 0.6–0.8. Bacterial cultures were centrifuged at 2500* g* for 10 min and resuspended in 10 mM MgCl_2_. The rhizosphere of Arabidopsis was inoculated three times over 3 weeks with 10 ml of OD_600_ = 0.1 using: *E.* *meliloti expR* + , *E.* *meliloti attM* culture solution or 10 mM MgCl_2_ as control. The production of AHL on the root surface as well as establishment of the bacteria was previously demonstrated by [45].

### Challenge with Pseudomonas syringae

*Pseudomonas syringae* pv. *tomato* DC3000 (*Pst*) pathogen assay on sterile Arabidopsis was performed as in Shrestha et al. [22]. The bacterial culture was adjusted to OD_600_ = 0.1. For *Pst* challenge on non-sterile condition, 3 days after the last inoculation with *E.* *meliloti* or MgCl_2_, four leaves from each treated plant were infiltrated with *Pst* inoculation solution adjusted to OD_600_ = 0.02. The leaves were infiltrated with *Pst* using a needleless syringe and four-leaf discs were collected from two leaves at 12, 48, and 96 h post infiltration. The leaf discs were homogenized in 10 mM MgCl_2_ and subsequently diluted. Duplicates of the dilution were plated on King’s B agar plates containing selective antibiotics to assess the colony-forming units (CFU) number.

### Assessment of localization of ALI1

To assess the subcellular localization of ALI1, *ALI1* ORF was subcloned into pGWB441 vector containing yellow fluorescent protein (YFP) gene and the Cauliflower Mosaic Virus (CaMV) 35S promoter and transformed into NEB 5-alpha Competent *E. coli* (New England Biolabs). Cloned vector extracted from transformed *E. coli* DH5α was used to transform competent *Agrobacterium tumefaciens* GV3101. YFP-tagged *ALI1* in *A. tumefaciens* GV3101 along with several *A.* *tumefaciens* LBA4404 containing mCherry-tagged gene sequence of proteins (Additional file 2: Table S3) that localizes at different subcellular organelles were grown overnight in 5 ml LB liquid medium with selective antibiotics at 28 °C. The bacterial cultures were centrifuged at 2500* g* for 10 min, resuspended with infiltration buffer at final OD_600_ of 0.05, and infiltrated into leaves of 4-week-old tobacco (*N. benthamiana*) plants. Equal OD_600_ 0.05 of *A.* *tumefaciens* GV3101 containing YFP-tagged *ALI1* and *A.* *tumefaciens* LBA4404 containing mCherry-tagged gene sequence of a protein that localizes at a subcellular organelle were mixed in infiltration buffer and infiltrated on the abaxial side of the leaves with 1-ml needleless syringe. The infiltrated leaves were visualized for protein localization on confocal laser scanning microscopy (CLSM) 2 to 3 days after infiltration. YFP was observed using 514 nm Ex and 520–550 nm Em range, and mCherry using 560 Ex and 570–600 Em settings.

### Localization of ALI1 upon oxo-C14-HSL treatment

In order to assess a possible relocalization of ALI1 upon oxo-C14-HSL treatment, the leaves that were pre-infiltrated for 24 h with *A. tumefaciens* mix solution were infiltrated again with 6 µM oxo-C14-HSL. Similarly, acetone on infiltration buffer was used as a control. The infiltrated leaves were visualized for ALI1 localization 1 and 2 days after the infiltration with oxo-C14-HSL.

### ALI1 expression and purification

Gateway destination vector pDEST17 subcloned with *ALI1* ORF was transformed into *E. coli* BL21 competent cells (New England Biolabs). Colonies were screened and selected with gene-specific and vector-specific primers (Additional file 2: Table S1). Positive colonies were grown in 50 ml of LB containing 100 mg ml^−1^ ampicillin at 37 °C until OD_600_ reached 0.6. The expression of the protein was induced by adding 1 mM isopropyl β-D-1-thiogalactopyranoside (IPTG) overnight at room temperature (RT). All further steps were carried out at 4 °C or on ice. Bacterial cells were collected and centrifuged at 7379* g* for 15 min and lysed through sonication with ice-cold lysis buffer (50 mM Tris–HCl, 300 mM NaCl, 0.1% Triton-X, DNase, lysozyme supplemented with protease inhibitor (Roche) and thereafter centrifuged at 14,462* g* for 10 min. The resulting supernatant was added to a solution containing nickel resin beads (HisPur™ Ni–NTA Resin, Thermo Fisher Scientific) and binding buffer (50 mM sodium phosphate buffer at pH 8, 500 mM NaCl, 10 mM imidazole, 2.5% glycerol) for 30 min with continuous rotation. Lysate-bead mix was subsequently transferred to column and washed 5 times with washing buffer (50 mM sodium phosphate buffer at pH 8, 500 mM NaCl, 20 mM imidazole, 2.5% glycerol) and eluted with elution buffer (50 mM sodium phosphate buffer at pH 8, 500 mM NaCl, 250 mM imidazole, 2.5% glycerol). The concentration of 6xHis-tagged proteins was quantified using Bradford assay (Roti-Quant, Roth). *E. coli* BL21 transformed with pDEST17-SpvC was used as a 6xHis-tagged protein control.

### Pull-down and binding assays

The interaction of ALI1 protein with oxo-C14-HSL was verified by pull-down assay. 6xHis-tagged ALI1 and 6xHis-tagged control protein were incubated with streptavidin beads (Sepharose® Bead Conjugate, Cell Signaling Technology) that were already loaded with M4 (oxo-C14-HSL tagged with biotin) or biotin control and incubated overnight before washing with washing buffer. The final bead-bound protein fractions were eluted with Laemmli buffer (62.5 mM Tris–HCl (pH 6.8), 2% SDS, 10% glycerol, 0.01% bromophenol blue) containing 2% β-mercaptoethanol and subjected to western blotting.

Thirty microliters of streptavidin beads were blocked using blocking buffer (0.05% (w/v) BSA in 100 mM phosphate buffer at pH 7.4) for half an hour at RT and further loaded with 60 mM biotin or 60 mM biotin-labeled oxo-C14-HSL (M4) for 30 min and centrifuged at 67* g* for 1 min. After removal of supernatant, 100 µg 6xHis-tagged proteins were added and incubated overnight at constant stirring at 4 °C. After the beads were washed five times with blocking buffer, bound proteins were eluted by adding 40 µl of Laemmli buffer and heated at 95 °C for 5 min. Twenty microliters of eluted protein samples was separated on SDS gel (12%) and transferred to PVDF membrane (Immun-Blot® PVDF, Bio-Rad) through semi-wet blotting protocol. The membranes were blocked with 5% fat-free milk and subsequently probed with primary antibodies 6xHis Tag Monoclonal Antibody (Invitrogen) followed by incubation with horseradish peroxidase-labeled secondary antibody. Blots were developed using ServaLight chemiluminescent substrate (SERVA).

To verify the binding capacity of ALI1 to oxo-C14-HSL, we used an additional indirect technique based on the detection of free oxo-C14-HSL by the bacterial biosensor *E. coil LuxCDABE*. We used BSA as a negative control and the *E. meliloti*-originated LuxR receptor as purified His and GST versions, as positive control, and the ALI1, purified as GST-ALI1 and 6xHis-ALI1 fusion proteins. Six nanomoles of each protein was used. The oxo-C14-HSL was added in a range from 0 to 18 nmol and incubated with proteins overnight. Unbound oxo-C14-HSL was detected after overnight incubation using the bacterial biosensor *E. coli LuxCDABE*, as described in Schenk et al. [33].

### Microscale thermophoresis (MST) binding assay

Preceding to MST bioassay, both proteins (6xHis-ALI1 and 6xHis-SpvC used as His-tagged protein control) were labeled with Red-Tris-NTA fluorescent dye and diluted into the labeling buffer (50 mM phosphate buffer at pH 8.0, 150 mM NaCl, 0.005% Tween-20, and 0.16% acetone) to reach the labeling concentration of 0.2 µM. After labeling, dye removal was not required as all dye molecules were bound by a protein due to labeling stoichiometry and, therefore, no free dye was present in the labeling batch. The final concentration of total protein and fluorescence was 200 and 100 nM respectively. Premium-coated capillaries were chosen for further experiments as no adhesion of both labeled proteins was observed. The intrinsic MST noise of both labeled proteins was acceptably low (< 5.0 units) under the applied experimental parameters and the used assay buffer. MST binding assay was performed on Monolith NT.115 Pico (red-nano) at 25 °C, with 90% LED power and 40% laser power. Five microliters of the fluorescent target proteins was used at constant 50 nM where 5 µl of the ligand oxo-C14-HSL was titrated from 100 µM down in 16 (1:1 dilution) steps. To ensure reproducibility, measurements were taken repeatedly on two independent experiments. The data were analyzed by ligand concentration (mol l^−1^) against normalized fluorescence of labeled proteins. Curve fitting were performed by using the K_D_ fit derived from the law of mass action according to the binding model, and the affinity is calculated and stated as EC50 or K_D_ values. The amplitude of the binding curve was assessed and signal to noise ratio which is amplitude divided by noise. Noise is standard deviation of difference between experimental data and fitted data.

### Nano-differential scanning fluorimetry (nanoDSF) assay

Prior to nanoDSF assay, both protein samples (6xHis-ALI1 and 6xHis-SpvC used as His-tagged protein control) and ligand oxo-C14-HSL were diluted in assay buffer (50 mM phosphate buffer at pH 8.0, 150 mM NaCl, 0.005% Tween-20 and 0.167% acetone) to have a final concentration of 5 and 100 µM respectively. The experiments were performed on a Prometheus NT.48 device equipped with additional back reflection options for detection of target protein aggregation via light scattering method. The temperature of the assay ranged from 20 to 95 °C with heating speed of 1 °C min^−1^. The analysis method of ratio 350/330 nm for protein unfolding (Tm) was used, and data were analyzed using the PR.StabilityAnalysissoftware from Nanotemper Technologies. The thermal denaturation of the target proteins were monitored via its intrinsic tryptophan fluorescence. Thermal shift was calculated by assessing difference of melting temperatures (Tm) between ligand-bound and apo-state of a protein.

### In silico screening for docking of oxo-C14-HSL against ALI1 (At5g14470)

The sequence of the ALI1 (AtGlcAK2) protein was obtained from TAIR, and the best predicted model of ALI1 was generated using i-TASSER server (https://zhanglab.ccmb.med.umich.edu/I-TASSER/) [51]. Similarly, 3D conformer of the ligand oxo-C14-HSL was retrieved from PubChem Structure [52]. The 3D structure of both protein and ligand was visualized using UCSF Chimera [53] where the model was prepared for docking in order to attain a clean protein model for further analysis. Docking simulations of ALI1 protein and oxo-C14-HSL ligand was performed in SwissDock (http://www.swissdock.ch/docking) [54], a web server that provides prediction of molecular interaction between the ligand and the target protein. The “docking type” was set to accurate type and “Definition of the region of interest” was set to default and so was “flexibility” which allows flexibility for side chains within 0 Å of any atom of the ligand in its reference binding mode. The binding modes were scored using their FullFitness and clustered, of which they were ranked on the basis of the average FullFitness of their elements [55]. The prediction file from SwissDock was further inspected in UCSF Chimera, and the chimera model with the lowest energy was selected for the identification of amino acid residues that were at a distance of less than 5 Å from each atom of the ligand.

### Cloning approach

*ALI1* ORF sequence (*At5g14470*) was amplified from cDNA of Col-0 Arabidopsis using ALI1-specific primers (Additional file 2: Table S1). Subsequently, *ALI1* ORF sequence was supplemented with *attB* sites using sequence-specific primers containing *attB* sites (Additional file 2: Table S1). Thereafter, the PCR product was purified using PEG precipitation protocol [56]. BP clonase recombination reaction was performed to subclone *ALI1* into the pDONR207 vector using Gateway® BP Clonase™ II Enzyme Mix following the manufacturer’s protocol. NEB 5-alpha Competent *E. coli* (New England Biolabs) cells were transformed with the product of recombination reaction using the heat shock method. Colony PCR was performed using DNR3 and DNR5 primers and amplicons were digested with BamHI and ran on a gel for further analysis. Positive constructs were sent for sequencing. LR clonase recombination reaction was performed to insert *ALI1* ORF into different destination vectors (pGWB21 or pGWB441) using Gateway™ LR Clonase™ II Enzyme Mix following the manufacturer’s protocol. Different destination vectors were used for the study. For protein expression and purification, pDEST17 was used whereas pGWB441 was used for cellular localization studies.

### Complementation of ali1 mutant

For complementation of the *ali1* mutant (N560407), pGWB21 (35S, N-ter 10xMyc) destination vector was used. Competent *E. coli* DH5α was transformed with the product of LR recombination reaction using heat shock method. Colony PCR was performed using vector and gene-specific primers (Additional file 2: Table S1) and obtained constructs were verified via sequencing. *A.* *tumefaciens* strain GV3101 was transformed with the desired vector constructs by electroporation, and selection of the positive colonies was performed through colony PCR using vector and gene-specific primers (Additional file 2: Table S1). The positive cells were used to transform the *ali1* mutant using the floral-dip method. Seeds of the *ali1* mutant were first surface sterilized and grown on ½-strength MS media plates for 2 weeks and later transferred on standard bedding soil (Fruhstorfer erde: Perlite (1:1)). The 2-week-old seedlings were grown on controlled condition (day/night 8/16 h and 22 °C photoperiod, light intensity of 150 µmol/ m^−2^ s^−1^ and 60% humidity) in a growth chamber for 2 months before transferring them to the greenhouse condition (day/night 16/8 h and 22 °C photoperiod, light intensity of 150 µmol m^−2^ s^−1^ and 60% humidity) until flowering. *A.* *tumefaciens* GV3101 strains containing *ALI1* ORF in pGWB21 was grown overnight in LB medium containing selective antibiotics. Two hundred fifty microliters of pre-culture was inoculated on 250 ml LB medium containing selective antibiotics and was grown overnight at 28 °C until the O.D_600_ reached 1. The culture was collected and centrifuged at 2500* g* for 10 min at RT. The pellets were resuspended in 250 ml of 5% sucrose solution, and Silwet L-77 was added at a concentration of 0.02%. Floral-dip method [57] was employed to stably transform the *ali1* mutant. Inflorescences were dipped into the *Agrobacterium* suspension for 30 s. The plants were then covered with a hood for 48 h and left to grow in the greenhouse under long-day conditions. Watering of the plants was stopped after the first pods began to dry. Seeds were harvested after complete drying of the inflorescences, and seeds were selected on selective ½-strength MS medium plates. The seedlings growing on selective plates were transferred on soil pots and later genotyped in order to identify the positive heterozygous complemented lines (*T*_0_). The positive complemented lines were self-pollinated and the generated seeds (F_1_) were checked for homozygosity using gene-specific and tag-specific primers (Additional file 2: Table S1).

### Western blot on complemented ali1 lines

Arabidopsis seedlings Col*-*0, complemented *ali1* (N560407) mutants #10–2 and #10–19, and outcross line #10–3 were first grown on ½-strength MS plates for 2 weeks and then transferred on soil pots. The plants were grown for additional 4 weeks and one leaf was collected and homogenized. Proteins were extracted from homogenized plant samples using Laemmli buffer (62.5 mM Tris–HCl (pH 6.8), 2% SDS, 10% glycerol, 0.01% bromophenol blue) further supplemented with Triton-X (10%) and additional SDS (4%). The homogenized plant samples were vortexed vigorously, subsequently cooked at 95 °C for 10 min and briefly centrifuged. Fifteen microliters of total protein was run on SDS gel (12%) and transferred to PVDF membrane through semi-wet blotting protocol. The membranes were blocked with 5% w/v fat-free milk and thereafter probed with primary antibody Myc-tag antibody (Chromotek GmbH), followed by incubation with horseradish peroxidase-labeled secondary antibody Anti-rat IgG (Cell Signaling Technology). Blots were developed using chemiluminescent substrate (ServaLight Vega Luminol solution, SERVA).

### Statistical analysis

All experiments were performed with at least three independent biological replicates. The GENMOD procedure from SAS 9.4 (SAS Institute Inc., Cary, NC, USA) was used for the analysis of variance. For multiple comparisons in root growth assay, the *p*-value was adjusted by the method of Tukey’s honestly significant difference (HSD) post hoc test. The class variable was treatment (C6-HSL, oxo-C14-HSL and acetone control). Quantitative PCR assays were performed in four biologically independent experiments and *Pst* assays were performed in three biologically independent experiments. *p* values < 0.05 in Student’s *t* test were considered as indicative for a significant difference. Western blot analysis was performed in three independent experiments, representative blot is shown.

## Supplementary Information

**Additional file 1. **Supporting individual data values displayed in Figure 1a, 1d, 3a-b, 5d, 6 a-d, 7a-b, and Additional file 2: Figure S1 and S3.

**Additional file 2: Supplementary materials. Figure S1.** The expression of *ALI1* is not influenced by oxo-C14-HSL. **Figure S2.** The *ali1* mutant has a T-DNA insertion in *At5g14470* position and is homozygous. **Figure S3.** Enhanced activation of defense-related genes due to AHL-priming is missing in *ali1*. **Figure S4.** ALI1 does not colocalize with Golgi, plastids, and peroxisomes. **Figure S5.** Localization of ALI1 upon plasmolysis. **Figure S6.** Representative images of predicted docking between ALI1 protein and oxo-C14-HSL ligand. **Figure S7.** Interaction between ALI1 and oxo-C14-HSL was missing in biophysical assays but indicated to be present in indirect binding assay. **Figure S8.** The complemented lines of *ali1* mutant express ALI1. **Table S1.** Densitometric analysis of Western blot results. **Table S2.** Primers used in the study. **Table S3.** Fluorescence-tagged strains used in the localization study.

**Additional file 3: Dataset S1.** List of genes differentially expressed (upregulated) in Col-0 after 3-day pretreatment with oxo-C14-HSL and additional challenge with 100 nM flg22 for 2 h.

**Additional file 4: Dataset S2.** List of genes differentially expressed (downregulated) in Col-0 after 3-day pretreatment with oxo-C14-HSL and additional challenge with 100 nM flg22 for 2 h.

**Additional file 5: Dataset S3.** List of genes differentially expressed (upregulated) in *ali1* after 3-day pretreatment with oxo-C14-HSL and additional challenge with 100 nM flg22 for 2 h.

**Additional file 6: Dataset S4. **List of genes differentially expressed (downregulated) in *ali1* after 3-day pretreatment with oxo-C14-HSL and additional challenge with 100 nM flg22 for 2 h.

## Data Availability

All data generated or analyzed during this study are included in this published article, its supplementary information files (Additional files 1, 2, 3, 4, 5 and 6) and publicly available repositories. The raw sequences of transcriptome analysis were uploaded to Gene Expression Omnibus (GEO) NCBI Sequence Read Archive with the accession number GSE156726 inhttps://www.ncbi.nlm.nih.gov/geo/query/acc.cgi?acc=GSE156726.

## Abbreviations

AHL: *N*-Acyl homoserine lactones
AtCaM: Calmodulin
AtGALK2: Galactokinase 2
AtGlcAK1: Glucurunokinase 1
AtGlcAK2: Glucurunokinase 2
BSA: Bovine serum albumin
C4-HSL: *N*-Butanoyl-*L*-homoserine lactone
C6-HSL: *N-*Hexanoyl-*L*-homoserine lactone
C10-HSL: *N*-Decanoyl-*L*-homoserine lactone
CFU: Colony-forming unit
CLSM: Confocal laser scanning microscopy
ER: Endoplasmic reticulum
GO: Gene Ontology
MST: Microscale thermophoresis
nanoDSF: Nano-differential scanning fluorimetry
oxo-C6-HSL: *N-*3-Oxo-hexanoyl-*L*-homoserine lactone
oxo-C8-HSL: *N-*3-Oxo-octanoyl-*L*-homoserine lactone
oxo-C10-HSL: *N-*3-Oxo-decanoyl-*L*-homoserine lactone
oxo-C14-HSL: *N-*3-Oxo-tetradecanoyl-*L*-homoserine lactone
Pst: *Pseudomonas syringae* Pv. *tomato*
PM: Plasma membrane
qPCR: Quantitative polymerase chain reaction
QS: Quorum sensing
SpvC: *Salmonella* Plasmid virulence C
YFP: Yellow fluorescent protein

## References

[1] Hense AH, Kuttler C, Müller J, Rothballer M, Hartmann A, Kreft J-U (2007). Opinion-does efficiency sensing unify diffusion and quorum sensing?. Nat Rev Microbiol.

[2] Ng WL, Bassler BL (2009). Bacterial quorum-sensing network architectures. Annu Rev Genet.

[3] Miller MB, Bassler BL (2001). Quorum sensing in bacteria. Annu Rev Microbiol.

[4] Geske GD, O'Neill JC, Blackwell HE (2008). Expanding dialogues: from natural autoinducers to non-natural analogues that modulate quorum sensing in Gram-negative bacteria. Chem Soc Rev.

[5] Ni N, Li M, Wang J, Wang B (2009). Inhibitors and antagonists of bacterial quorum sensing. Med Res Rev.

[6] Whitehead NA, Barnard AML, Slater H, Simpson NJL, Salmond GPC (2001). Quorum-sensing in Gram-negative bacteria. FEMS Microbiol Rev.

[7] Marketon MM, Gronquist MR, Eberhard A, Gonzalez JE (2002). Characterization of the *Sinorhizobium meliloti sinR/sinI* locus and the production of novel *N*-acyl homoserine lactones. J Bacteriol.

[8] von Bodman SB, Bauer WD, Coplin DL (2003). Quorum sensing in plant-pathogenic bacteria. Annu Rev Phytopathol.

[9] Williams P (2007). Quorum sensing, communication and cross-kingdom signalling in the bacterial world. Microbiology.

[10] Mathesius U, Mulders S, Gao M, Teplitski M, Caetano-Anolle's G, Rolfe BG (2003). Extensive and specific responses of a eukaryote to bacterial quorum-sensing signals. PNAS.

[11] Schenk ST, Stein E, Kogel KH, Schikora A (2012). Arabidopsis growth and defense are modulated by bacterial quorum sensing molecules. Plant Signal Behav.

[12] von Rad U, Klein I, Dobrev PI, Kottova J, Zazimalova E, Fekete A (2008). Response of *Arabidopsis thaliana* to *N*-hexanoyl-DL-homoserine-lactone, a bacterial quorum sensing molecule produced in the rhizosphere. Planta.

[13] Ortiz-Castro R, Martinez-Trujillo M, Lopez-Bucio J (2008). *N*-acyl-L-homoserine lactones: a class of bacterial quorum-sensing signals alter post-embryonic root development in *Arabidopsis thaliana*. Plant Cell Environ.

[14] Palmer AG, Senechal AC, Mukherjee A, Ane JM, Blackwell HE (2014). Plant responses to bacterial N-acyl L-homoserine lactones are dependent on enzymatic degradation to L-homoserine. ACS Chem Biol.

[15] Bai X, Todd CD, Desikan R, Yang Y, Hu X (2011). *N*-3-Oxo-Decanoyl-L-homoserine-lactone activates auxin-induced adventitious root formation via hydrogen peroxide- and nitric oxide-dependent cyclic GMP signaling in mung bean. Plant Physiol.

[16] Moshynets OV, Babenko LM, Rogalsky SP, Iungin OS, Foster J, Kosakivska IV (2019). Priming winter wheat seeds with the bacterial quorum sensing signal *N*-hexanoyl-L-homoserine lactone (C6-HSL) shows potential to improve plant growth and seed yield. PLoS ONE.

[17] Rankl S, Gunsé B, Sieper T, Schmid C, Poschenrieder C, Schröder P (2016). Microbial homoserine lactones (AHLs) are effectors of root morphological changes in barley. Plant Sci.

[18] Pazarlar S, Cetinkaya N, Bor M, Kara RS. *N*-acyl homoserine lactone-mediated modulation of plant growth and defense against *Pseudoperonospora cubensis* in cucumber (*Cucumis sativus* L.). J Exp Bot. 2020;71(20):6638–6654. 10.1093/jxb/eraa384 32822478

[19] Liu F, Zhao Q, Jia Z, Song C, Huang Y, Ma H (2020). *N*-3-oxo-octanoyl-homoserine lactone-mediated priming of resistance to *Pseudomonas syringae* requires the salicylic acid signaling pathway in *Arabidopsis thaliana*. BMC Plant Biol.

[20] Schikora A, Schenk ST, Stein E, Molitor A, Zuccaro A, Kogel KH (2011). *N*-acyl-homoserine lactone confers resistance toward biotrophic and hemibiotrophic pathogens via altered activation of AtMPK6. Plant Physiol.

[21] Hernández-Reyes C, Schenk ST, Neumann C, Kogel KH, Schikora A (2014). *N*-acyl-homoserine lactones-producing bacteria protect plants against plant and human pathogens. Microb Biotechnol.

[22] Shrestha A, Grimm M, Ojiro I, Krumwiede J, Schikora A. Impact of quorum sensing molecules on plant growth and immune system. Front Microbiol. 2020;11:1545. 10.3389/fmicb.2020.01545. 10.3389/fmicb.2020.01545 PMC7378388 32765447

[23] Barriuso J, Solano BR, Mañero FJG (2008). Protection against pathogen and salt stress by four plant growth-promoting Rhizobacteria isolated from Pinus sp. on Arabidopsis thaliana. Phytopathology..

[24] Zhao Q, Yang XY, Li Y, Liu F, Cao XY, Jia ZH (2020). N-3-oxo-hexanoyl-homoserine lactone, a bacterial quorum sensing signal, enhances salt tolerance in Arabidopsis and wheat. Bot Stud.

[25] Götz-Rösch C, Sieper T, Fekete A, Schmitt-Kopplin P, Hartmann A, Schröder P (2015). Influence of bacterial *N*-acyl-homoserine lactones on growth parameters, pigments, antioxidative capacities and the xenobiotic phase II detoxification enzymes in barley and yam bean. Front Plant Sci.

[26] Gupta GS, Kumar A, Verma N (2019). Bacterial homoserine lactones as a nanocomposite fertilizer and defense regulator for chickpeas. Environ Sci : Nano.

[27] Song S, Jia Z, Xu J, Zhang Z, Bian Z (2011). *N*-butyryl-homoserine lactone, a bacterial quorum-sensing signaling molecule, induces intracellular calcium elevation in *Arabidopsis* root cells. Biochem Biophys Res Commun.

[28] Zhao Q, Zhang C, Jia Z, Huang Y, Li H, Song S (2015). Involvement of calmodulin in regulation of primary root elongation by *N*-3-oxo-hexanoyl homoserine lactone in *Arabidopsis thaliana*. Front Plant Sci.

[29] Zhao Q, Li M, Jia Z, Liu F, Ma H, Huang Y (2016). AtMYB44 positively regulates the enhanced elongation of primary roots induced by *N*-3-Oxo-Hexanoyl-homoserine lactone in *Arabidopsis thaliana*. Mol Plant Microbe Interact.

[30] Liu F, Bian Z, Jia Z, Zhao Q, Song S (2012). The GCR1 and GPA1 participate in promotion of *Arabidopsis* primary root elongation induced by *N*-Acyl-Homoserine lactones, the bacterial Quorum-sensing signals. MPMI.

[31] Götz C, Fekete A, Gebefuegi I, Forczek ST, Fuksova K, Li X (2007). Uptake, degradation and chiral discrimination of *N*-acyl-D/L-homoserine lactones by barley (*Hordeum vulgare*) and yam bean (*Pachyrhizus erosus*) plants. Anal Bioanal Chem.

[32] Sieper T, Forczek S, Matucha M, Kramer P, Hartmann A, Schroder P (2014). *N*-acyl-homoserine lactone uptake and systemic transport in barley rest upon active parts of the plant. New Phytol.

[33] Schenk ST, Hernandez-Reyes C, Samans B, Stein E, Neumann C, Schikora M (2014). *N*-Acyl-homoserine lactone primes plants for cell wall reinforcement and induces resistance to bacterial pathogens via the salicylic acid/oxylipin pathway. Plant Cell.

[34] Schenk ST, Schikora A (2014). AHL-priming functions via oxylipin and salicylic acid. Front Plant Sci.

[35] Shrestha A, Elhady A, Adss S, Wehner G, Böttcher C, Heuer H (2019). Genetic differences in barley govern the responsiveness to *N*-Acyl homoserine lactone. Phytobiomes Journal.

[36] Nelson BK, Cai X, Nebenfuhr A (2007). A multicolored set of *in vivo* organelle markers for co-localization studies in Arabidopsis and other plants. Plant J.

[37] Thomanek H, Schenk ST, Stein E, Kogel KH, Schikora A, Maison W (2013). Modified *N*-acyl-homoserine lactones as chemical probes for the elucidation of plant-microbe interactions. Org Biomol Chem.

[38] Egert A, Peters S, Guyot C, Stieger B, Keller F (2012). An Arabidopsis T-DNA insertion mutant for galactokinase (*AtGALK*, At3g06580) hyperaccumulates free galactose and is insensitive to exogenous galactose. Plant Cell Physiol.

[39] Pieslinger AM, Hoepflinger MC, Tenhaken R (2010). Cloning of Glucuronokinase from Arabidopsis thaliana, the last missing enzyme of the myo-inositol oxygenase pathway to nucleotide sugars. J Biol Chem.

[40] Ivanov Kavkova E, Blochl C, Tenhaken R (2019). The Myo-inositol pathway does not contribute to ascorbic acid synthesis. Plant Biol (Stuttg).

[41] Zhao Q, Yu D, Chang H, Guo X, Yuan C, Hu S (2013). Regulation and function of *Arabidopsis AtGALK2* gene in abscisic acid response signaling. Mol Biol Rep.

[42] Widmann C, Gibson S, Jarpe MB, Johnson GL (1999). Mitogen-activated protein kinase: conservation of a three-kinase module from yeast to human. Physiol Rev.

[43] Davis S, Vanhoutte P, Pagès C, Caboche J, Laroche S (2000). The MAPK/ERK cascade targets both Elk-1 and cAMP response element-binding protein to control long-term potentiation-dependent gene expression in the dentate gyrus *in vivo*. J Neurosci.

[44] Beckers GJ, Jaskiewicz M, Liu Y, Underwood WR, He SY, Zhang S (2009). Mitogen-activated protein kinases 3 and 6 are required for full priming of stress responses in *Arabidopsis thaliana*. Plant Cell.

[45] Zarkani AA, Stein E, Rohrich CR, Schikora M, Evguenieva-Hackenberg E, Degenkolb T (2013). Homoserine lactones influence the reaction of plants to Rhizobia. Int J Mol Sci.

[46] Bayer EM, Sparkes I, Vanneste S, Rosado A (2017). From shaping organelles to signalling platforms: the emerging functions of plant ER-PM contact sites. Curr Opin Plant Biol.

[47] Hilson P, Allemeersch J, Altmann T, Aubourg S, Avon A, Beynon J (2004). Versatile gene-specific sequence tags for Arabidopsis functional genomics: transcript profiling and reverse genetics applications. Genome Res.

[48] Dobin A, Davis CA, Schlesinger F, Drenkow J, Zaleski C, Jha S (2013). STAR: ultrafast universal RNA-seq aligner. Bioinformatics.

[49] Trapnell C, Williams BA, Pertea G, Mortazavi A, Kwan G, van Baren MJ (2010). Transcript assembly and quantification by RNA-Seq reveals unannotated transcripts and isoform switching during cell differentiation. Nat Biotechnol.

[50] Larsson J. eulerr: area-proportional euler and venn diagrams with ellipses. R package version 6.1.0 ed: Larsson, J.; 2020.

[51] Yang J, Zhang Y. Protein structure and function prediction using I-TASSER. Curr Protoc Bioinformatics. 2015;52:5 8 1–5 8 15. 10.1002/0471250953.bi0508s52 PMC4871818 26678386

[52] Kim S, Thiessen PA, Bolton EE, Chen J, Fu G, Gindulyte A (2016). PubChem substance and compound databases. Nucleic Acids Res.

[53] Pettersen EF, Goddard TD, Huang CC, Couch GS, Greenblatt DM, Meng EC (2004). UCSF Chimera–a visualization system for exploratory research and analysis. J Comput Chem.

[54] Grosdidier A, Zoete V, Michielin O (2011). SwissDock, a protein-small molecule docking web service based on EADock DSS. Nucleic Acids Res..

[55] Grosdidier A, Zoete V, Michielin O (2007). EADock: docking of small molecules into protein active sites with a multiobjective evolutionary optimization. Proteins.

[56] Schmitz A, Riesner D (2006). Purification of nucleic acids by selective precipitation with polyethylene glycol 6000. Anal Biochem.

[57] Clough SJ, Bent AF (1998). Floral dip: a simplified method for *Agrobacterium*-mediated transformation of *Arabidopsis thaliana*. Plant J.

