# Supplementary material for: AHL-Priming Protein 1 mediates N-3-oxo-tetradecanoyl-homoserine lactone priming in Arabidopsis
Source: BMC Biol. 2022 Dec 5;20:268. doi: 10.1186/s12915-022-01464-3 (PMC9721052; doi:10.1186/s12915-022-01464-3)
Supplement: Supplementary file 2 — Additional file 2: Supplementary materials. Figure S1. The expression of ALI1 is not influenced by oxo-C14-HSL. Figure S2. The ali1 mutant has a T-DNA insertion in At5g14470 position and is homozygous. Figure S3. Enhanced activation of defense-related genes due to AHL-priming is missing in ali1. Figure S4. ALI1 does not colocalize with Golgi, plastids, and peroxisomes. Figure S5. Localization of ALI1 upon plasmolysis. Figure S6. Representative images of predicted docking between ALI1 protein and oxo-C14-HSL ligand. Figure S7. Interaction between ALI1 and oxo-C14-HSL was missing in biophysical assays but indicated to be present in indirect binding assay. Figure S8. The complemented lines of ali1 mutant express ALI1. Table S1. Densitometric analysis of Western blot results. Table S2. Primers used in the study. Table S3. Fluorescence-tagged strains used in the localization study. [file 12915_2022_1464_MOESM2_ESM.pdf]

Additional file 2: Supplementary materials for

AHL-Priming Protein1 mediates *N*-3-oxotetradecanoyl-homoserine lactone priming in Arabidopsis

Abhishek Shrestha[1], Casandra Hernández-Reyes[2], Maja Grimm[1], Johannes Krumwiede[1], Elke Stein[3], Sebastian T. Schenk[2] and Adam Schikora[1]*

*Corresponding authors:
Adam Schikora, email: adam.schikora@julius-kuehn.de

The following Supporting Information is available for this article:

Fig. S1 The expression of *ALI1* is not influenced by oxo-C14-HSL.

Fig. S2 The *ali1* mutant has a T-DNA insertion in *At5g14470* position and is homozygous.

Fig. S3 Enhanced activation of defense-related genes due to AHL-priming is missing in *ali1*.

Fig. S4 ALI1 does not colocalize with Golgi, plastids, and peroxisomes.

Fig. S5 Localization of ALI1 upon plasmolysis.

Fig. S6 Representative images of predicted docking between ALI1 protein and oxo-C14-HSL ligand.

Fig. S7 Interaction between ALI1 and oxo-C14-HSL was missing in biophysical.

Fig. S8 The complemented lines of *ali1* mutant express ALI1.

Table S1 Densitometric analysis of Western blot results

Table S2 Primers used in the study.

Table S3 Fluorescence-tagged strains used in the localization study.

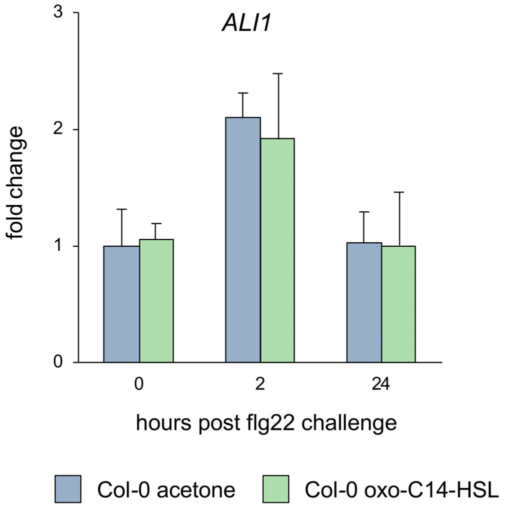

Fig. S1 The expression of *ALI1* is not influenced by oxo-C14-HSL. Expression profile of *ALI1* was monitored at three time points after challenge with 100 nM flg22 in *Arabidopsis* wild-type Col-0. Plants were grown on sterile hydroponic system and pretreated with 6 µM oxo-C14-HSL or acetone (solvent control) three days prior to flg22 challenge. The abundance of each gene transcript was normalized with *Ubiquitin ligase* (*At5g25760*) transcript and 0 hpt (hours post treatment) levels. The bar represents mean and error bars SD from four independent biological replicates, individual data values for S1 in Additional File 1.

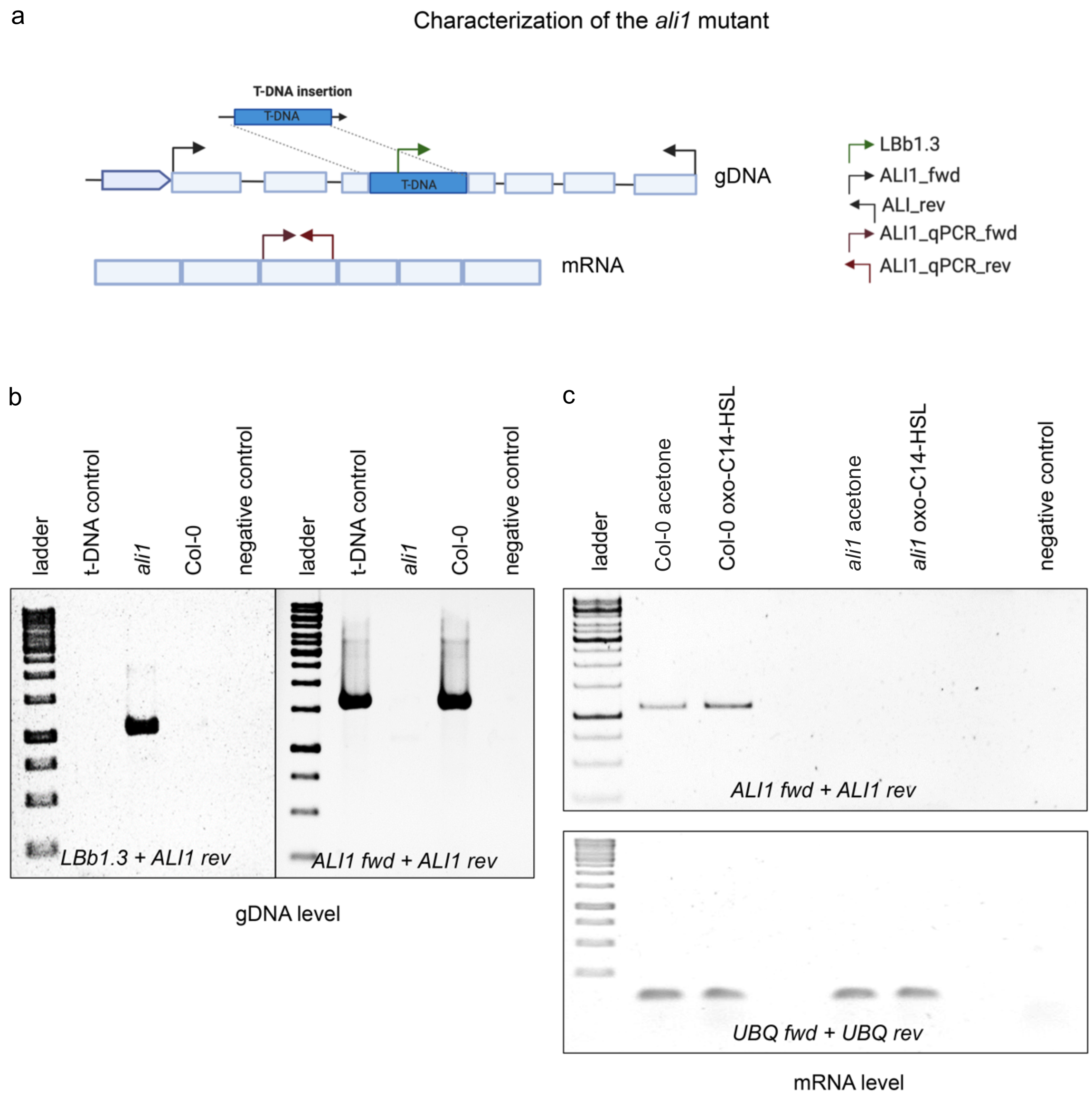

Fig. S2 The *ali1* mutant has a T-DNA insertion in *At5g14470* position and is homozygous. **a** Graphical representation of T-DNA insertion in *ALI1* gene, indicating primers (Additional file 2: Table S1) used for characterization of the mutant. **b** PCR-based characterization of the genetical structure of *ali1*. The insertion of T-DNA and the absence of functional *ALI1* gene in *ali1* mutant however, absence of T-DNA insertion and the presence of functional *ALI1* gene in wild-type Col-0 were evidenced by PCR products using primers as indicated. **c** The expression of *ALI1* in both acetone and oxo-C14-HSL-pretreated Col-0 plants as well as the absence of *ALI1* expression in both acetone and oxo-C14-HSL-pretreated *ali1* mutant, as evidenced by PCR approach. The expression of housekeeping gene *Ubiquitin ligase* (*UBQ*) in both acetone and oxo-C14-HSL-pretreated Col-0 and *ali1* mutant. T-DNA left border-specific LBb1-3 and *ALI1*-specific reverse primers were used to detect the presence of T-DNA insertion in *ALI1* gene, whereas gene-specific primers, ALI1 fwd and ALI1 rev were used in a separate reaction to detect the presence of *ALI1* gene in both, DNA and mRNA levels and *UBQ* gene-specific primers, UBQ fwd and UBQ rev were used in a separate reaction for the internal control.

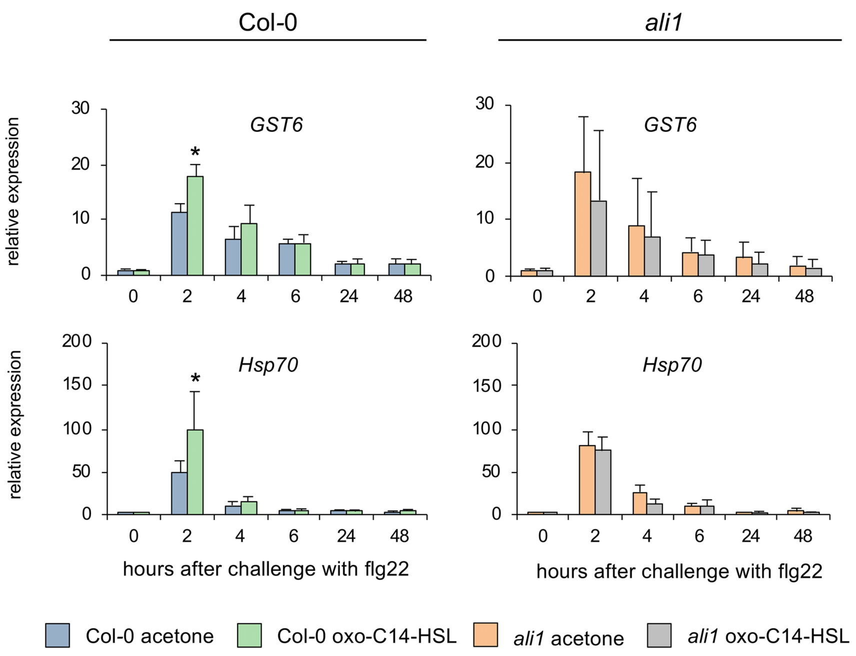

Fig. S3 Enhanced activation of defense-related genes due to AHL-priming is missing in *ali1*. Expression profile of defense-related genes *GST6* and *Hsp70* was monitored at various time points after 100 nM flg22 challenge in Col-0 and *ali1* mutant. Plants were grown on a sterile hydroponic system and pretreated with 6 µM oxo-C14-HSL or acetone (solvent control) for three days prior to flg22 challenge. The abundance of each gene transcript was normalized with *Ubiquitin ligase* (*At5g25760*) transcript and 0 hpt (hours post treatment) levels. Error bars represent SD from four independent biological repetitions. * indicates $p < 0.05$ in Student's *t*-test, individual data values for S3 in Additional File 1.

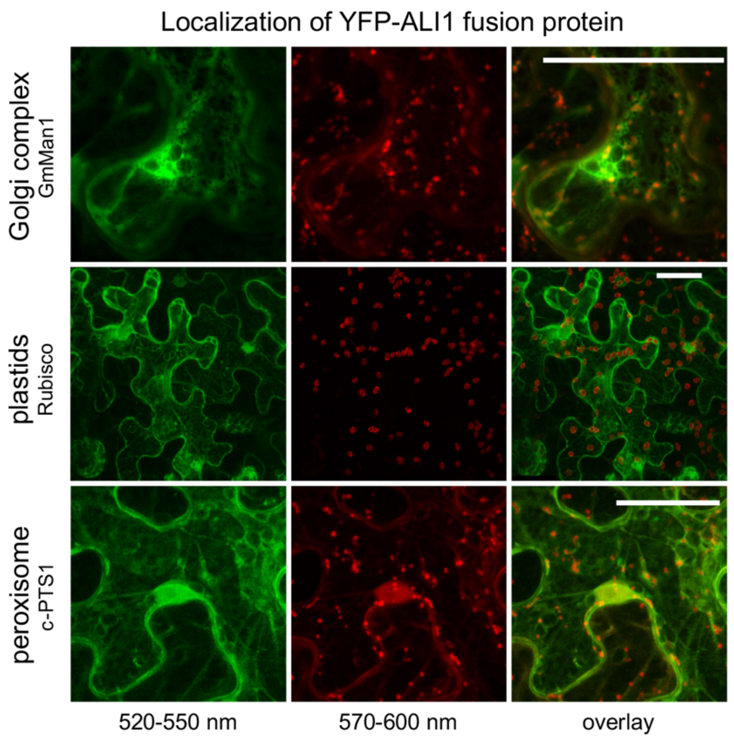

Fig. S4 ALI1 does not colocalize with Golgi, plastids, and peroxisomes. Plasmids carrying YFP-tagged ALI1 version and mCherry-marked proteins localizing to Golgi (GmMan1), plastids (RuBisCO) or peroxisome (PTS1) were co-transformed into *N. benthamiana* leaf epidermal cells by *Agrobacterium*-infiltration. The cells were analyzed 2 days after the infiltration using CLSM. The left panel shows in green, the fluorescence of the YFP-tagged ALI1, whereas the middle panel shows in red, the fluorescence of different mCherry-marked proteins colocalizing to different subcellular compartments (Additional file 2: Table S2). The right panel shows the corresponding merged image, where yellow color indicates a colocalization. Scale bar = 40 μm.

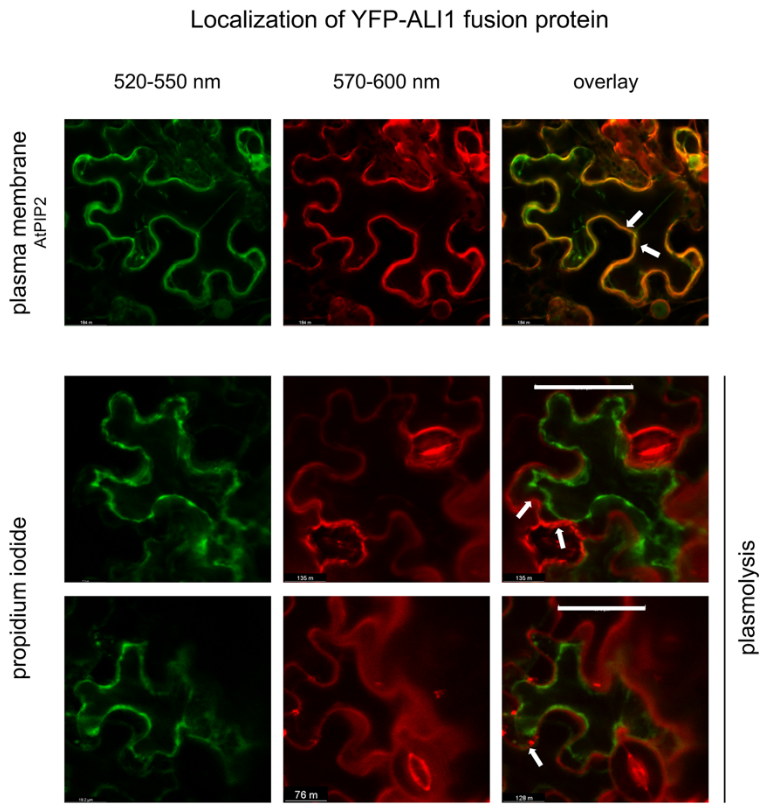

Fig. S5 Localization of ALI1 upon plasmolysis. Plasmids carrying YFP-tagged ALI1 version and mCherry-marked proteins localizing in PM (AtPIP2) were co-transformed to *N. benthamiana* leaf epidermal cells by *Agrobacterium*-infiltration. The cells were analyzed 2 days after the infiltration. The left panel shows in green the fluorescence of YFP-tagged ALI1 version, whereas the middle panel shows in red fluorescence of different mCherry-marked subcellular localizing proteins (upper panel), or the fluorescence of the propidium iodide. The right panel shows corresponding merged images where yellow color indicates colocalization. Scale bar = 40 µm. Arrows indicate the plasmolysis site (lower panels) or co-localization with AtPIP2 (upper panel).

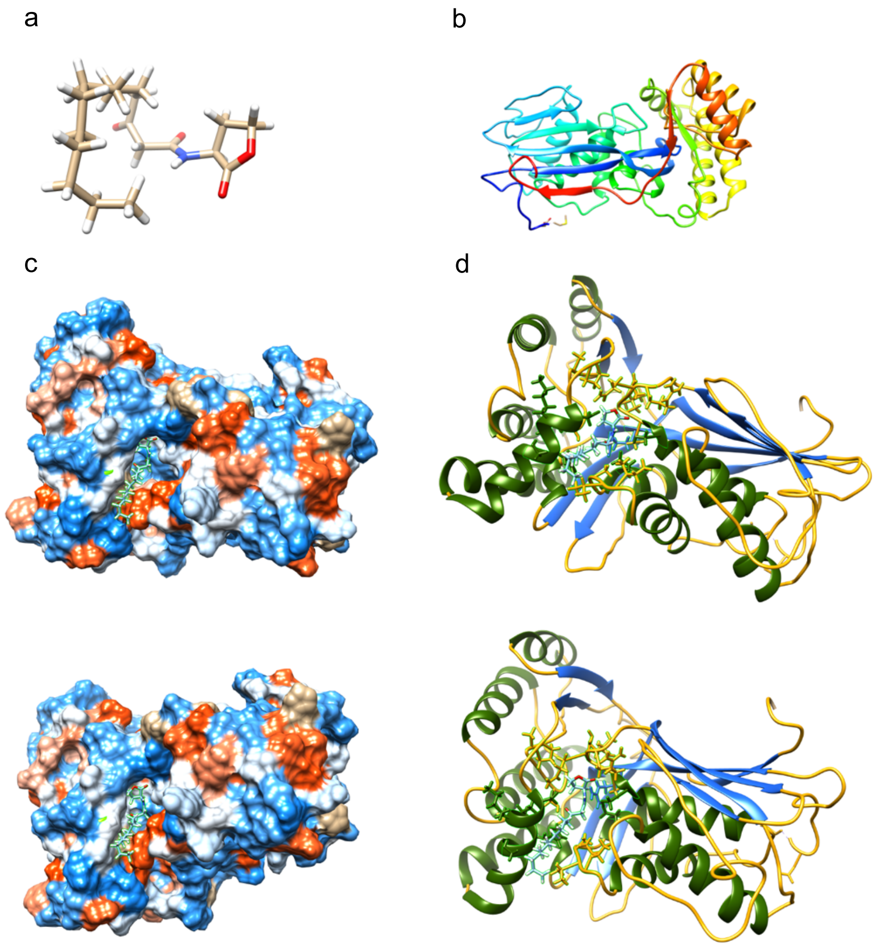

e

MDPNPKPAISGKDNGVFEHRSFA**R**IGFLGNPSDVYFGRTISFTIGNFWAWAKLEPSDHL
LIKPHPFHDLVQFDSLDNLVYRLENDGYYGGVRLLMAICKVFRNYCKENGIQLHDKNFT
LSYDTNIP**RQTGLSGS**SAIVSAALSCLLDFYNVRQSIRIEVRPNLILNAEKEL**GIVAG**LQ**D**
RVAQVYGGGLVHMDFSKEHMDKVGYGIYTIMDINLLPPLHLIYAENPSD**SGKVHS**TVRR
RWLDGDEFIISSMAEIAKLAEEGRTALLKKDYSNLKELMNRNFDLRRSMFGDECLGAM
NIEMVEVARKIGAAAKFT**GS**GG**A**VVVFCPEGPSQVKLLEEECRKSGFIVEPVKLVPTRL
SSSDIKTLSKT

Fig. S6 Representative images of predicted docking between ALI1 protein and oxo-C14-HSL ligand. The tertiary structure of (**a**) *N*-3-oxotetradecanoyl-*L*-homoserine lactone (oxo-C14-HSL) and (**b**) the predicted structure of ALI1 protein. The predicted hydrophobicity (**c**) and ribbon (**d**) structure of the ALI1 protein with the oxo-C14-HSL ligand from different angles. **e** Amino acid sequences of ALI1, bold letters indicate residues with predicted distance of less than 5 Å from the ligand.

a

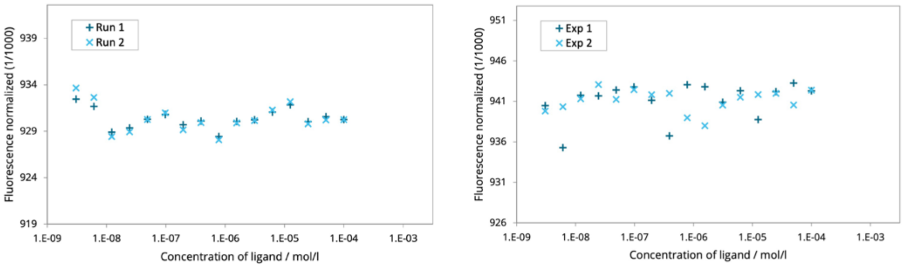

	K_D	Amplitude	S/N	
**Run 1**	---	---	---	---
**Run 2**	---	---	---	---

	K_D	Amplitude	S/N	
**Run 1**	---	---	---	---
**Run 2**	---	---	---	---

b

Annotations				Ratio ↗								Scattering ↗	
				IP #1		Δ IP #1		IP #2		Δ IP #2		ON	
Sample ID	µM_protein	ligand	µM_ligand	⌀	σ	⌀	σ	⌀	σ	⌀	σ	⌀	σ
6xHis-ALI1	5	-	-	50.88	0.17	-	-	66.51	0.81	-	-	45.52	0.79
6xHis-ALI1	5	oxo-C14-HSL	100	48.97	0.20	-1.91	0.26	65.63	0.06	-0.88	0.81	45.07	0.11

Annotations				Ratio ↗								Scattering ↗	
				IP #1		Δ IP #1		IP #2		Δ IP #2		ON	
Sample ID	µM_protein	ligand	µM_ligand	⌀	σ	⌀	σ	⌀	σ	⌀	σ	⌀	σ
6xHis-SpvC	5	-	-	43.99	0.03	-	-	-	-	-	-	39.07	0.79
6xHis-SpvC	5	oxo-C14-HSL	100	43.19	0.02	-0.80	0.03	-	-	-	-	39.11	0.08

Fig. S7 Interaction between ALI1 and oxo-C14-HSL was missing in biophysical assays. **a** MST binding assay missing to quantify the interaction study between fluorescently labeled proteins 6xHis-ALI1 (left) or 6xHis-SpvC (right) and ligand oxo-C14-HSL. The protein concentration was kept constant at 50 nM and the ligand oxo-C14-HSL was titrated from 100 µM to 3.05 nM. The difference in normalized fluorescence (%) was plotted for analysis of thermophoresis. Two experimental replicates were used for the analysis. **b** Nano-DSF assay suggesting comparable thermal shifts in both proteins 6xHis-AI1 and 6xHis-SpvC in the presence of ligand oxo-C14-HSL which could possibly be an artifact. Both proteins and ligand were diluted to have a final concentration of 5 µM and 100 µM respectively. For both proteins, unfolding transition was assessed. Thermal shift was calculated by assessing difference of melting temperatures (Tm) between ligand-bound and apo-state of a protein. Two experimental replicates were used for the analysis.

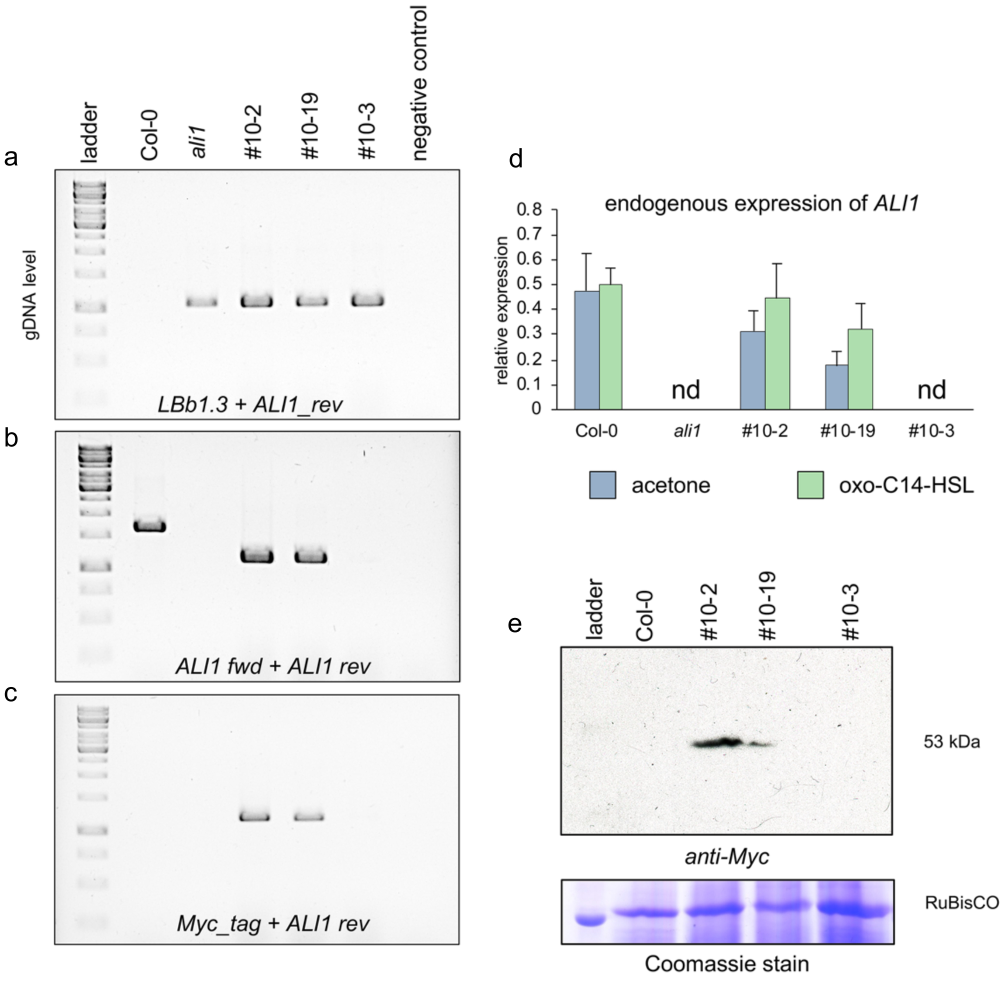

Fig. S8 The complemented lines of *ali1* mutant express ALI1. Ethidium bromide stained gel indicating: (**a**) The presence of T-DNA insertion in *ALI1* gene of *ali1* mutant, two complemented lines of *ali1*: #10-2 and #10-19, and outcrossed line #10-3 but not in wild-type Col-0; (**b**) The presence of *ALI1* gene in Col-0 (genomic version) and two complemented lines of *ali1*: #10-2 and #10-19 (mRNA version); and (**c**) The presence of *ALI1* gene tagged with 10xMyc in two complemented lines of *ali1*: #10-2 and #10-19. Primers T-DNA left border specific LBb1-3 and ALI1 rev were used to detect the presence of T-DNA insertion in *ALI1* gene, gene-specific primers ALI1 fwd and ALI1 rev were used to detect the presence of *ALI1* gene and tag-specific primer Myc-tag and ALI1 rev were used to verify the presence of Myc-tagged *ALI1* gene. **d** The endogenous expression level of *ALI1* in acetone and oxo-C14-HSL pretreated Col-0, *ali1*, two complemented lines of *ali1*: #10-2 and #10-19 and outcrossed line #10-3. Two-week old seedlings were transferred to ½-strength MS liquid medium supplemented with 6 µM oxo-C14-HSL or acetone control for three days. The abundance of *ALI1* gene transcript was normalized with *Ubiquitin ligase* (*At5g25760*) transcript. The bar represents mean and SD of three biological replicates. **e** Western blot indicating the expression of Myc-tagged ALI1 in both complemented lines #10-2 and #10-19 but not in outcross line #10-3. Representative blot is shown.

Table S1 Densitometric analysis of Western blot results

Quantification of Western blot results of phosphorylation activation of MAP kinase (Fig. 1b) by densitometry using ImageJ software.

		**Col-0**		***ali1***	
**Treatment**	**min post flg22**	***anti-AtMPK6***	***anti-pERK1/2***	***anti-AtMPK6***	***anti-pERK1/2***
acetone	0	83894.973	0	78646.822	0
	30	89831.416	81804.019	91850.115	84459.295
	60	76937.194	35286.999	75723.186	36074.475
	120	65882.123	0	84097.964	0
oxo-C14-HSL	0	79680.044	0	79905.843	0
	30	76780.467	73565.312	79181.408	74476.324
	60	72056.952	59484.948	87524.156	29908.919
	120	83795.437	25808.513	95755.42	0

Table S2 Primers used in the study

Oligonucleotide	Sequence (5´–3´)	Reference
ALI1 fwd	GGA GAT AGA ACC ATG GAT CCG AAT CCT AAA CCG	This study
ALI1 rev	TCC ACC TCC GGA TCM TGT TTT TGA TAA TGT CTT AAT ATC AGA AC	This study
U5 fwd	GGG GAC AAG TTT GTA CAA AAA AGC AGG CTT CGA AGG AGA TAG AAC CAT G	ATOME Project
U3 rev	AGA TTG GGG ACC ACT TTG TAC AAG AAA GCT GGG TCT CCA CCT CCG GAT C	ATOME Project
DNR5	CTG GCA GTT CCC TAC TCT CG	[47]
DNR3	GAT GGT CGG AAG AGG CAT AA	[47]
GTW1	TAG CTT CCT TAG CTC CTG AAA ATC TCG	ATOME Project
GTW2	GGG AAT ATA AAT GTC AGG CTC CCT TA	ATOME Project
35S-promoter	TTC GCA AGA CCC TTC CTC TAT A	universal
Actin fwd	GGT CGT ACA ACC GGT ATT GTG CTG G	C. Forzani (*personal comm.)*
Actin rev	TTG GAG ATC CAC ATC TGC TGG AAT G	C. Forzani (*personal comm.)*
T7	TAA TAC GAC TCA CTA TAG GG	universal
UBQ fwd	GCT TGG AGT CCT GCT TGG ACG	[33]
UBQ rev	CGC AGT TAA GAG GAC TGT CCG GC	
WRK22 fwd	ATC TCC GAC GAC CAC TAT TG	[33]
WRK22 rev	TCA TCG CTA ACC ACC GTA TC	
WRK29 fwd	TCC GGT ACG TTT TCA CCT TC	[33]
WRK29 rev	AGA GAC CGA GCT TGT GAG GA	
GST6 fwd	GCA TGT TCG GCA TGA CCA CTG	[33]
GST6 rev	GCA CCT TGG AGT CAG TAC CC	
Hsp70 fwd	CGC CAA CGA TCA AGG CAA CC	[33]
Hsp70 rev	GCT TCT CAC CTG GAC CGG AA	
ALI1 ins1	GAT CTT ACG TGC CAC TTC CAC	This study
ALI1 ins2	TTT TGA TTG AAA GAT TTG TGG C	This study
ALI1 qPCR fwd	GAC GGG GCT TTC AGG TTC TA	This study
ALI1 qPCR rev	AGA CTT GAG CAA CAC GGT CT	This study
LBb1-3	ATT TTG CCG ATT TCG GAA C	Salk Institute
Myc-tag	AAT CTC CGA GGA AGA CTT GAA C	This study
TLP5 fwd	GAAAGAGGAAGAAGGAAAGGT	This study
TLP5 rev	CAGAAGCTACAGTCACTCTC	This study
DFR fwd	CAAACGCCAAGACGCTACTCAC	This study
DFR rev	ACGGTCTTTGCCTTAACACATGC	This study

Table S3 Fluorescence-tagged strains used in the localization study
Localization markers used in this study in the *Agrobacterium*-mediated transformation.

Strain	Remarks	Localization	Reference
A. tumefaciens (LBA4404)	pBin20 containing mCherry with the ATWAK2 signal peptide and HDEL-motif	endoplasmic reticulum	[36]
A. tumefaciens (LBA4404)	pBin20 containing mCherry fused to full-length coding region of AtPIP2A, a plasma membrane aquaporin (Cutler et al., 2000)	plasma membrane	[36]
A. tumefaciens (LBA4404)	pBin20 containing mCherry fused to the C-terminus of c-TIP, an aquaporin of the vacuolar membrane (Saito et al., 2002)	tonoplast	[36]
A. tumefaciens (LBA4404)	pBin20 containing mCherry fused to peroxisomal targeting signal1 (PTS1, Ser-Lys-Leu) at its C-terminus	peroxisome	[36]
A. tumefaciens (LBA4404)	pBin20 containing mCherry fused to first 29 aa of yeast (*Saccharomyces cerevisiae*) cytochrome c oxidase IV	mitochondria	[36]
A. tumefaciens (LBA4404)	pBin20 containing mCherry fused to targeting sequence (first 79 aa) of the small subunit of tobacco rubisco	plastids	[36]
A. tumefaciens (LBA4404)	pBin20 containing mCherry fused to cytoplasmic tail and transmembrane domain (first 49 aa) of GmMan1, soybean a-1,2-mannosidase I	Golgi	[36]
